# Oxidative Stress in Neurodegenerative Diseases

**DOI:** 10.1007/s12035-015-9337-5

**Published:** 2015-07-22

**Authors:** Ewa Niedzielska, Irena Smaga, Maciej Gawlik, Andrzej Moniczewski, Piotr Stankowicz, Joanna Pera, Małgorzata Filip

**Affiliations:** 1 Department of Toxicology, Chair of Toxicology, Faculty of Pharmacy, Jagiellonian University, Medical College, Medyczna 9, 30-688 Kraków, Poland; 2 Department of Neurology, Faculty of Medicine, Jagiellonian University, Medical College, Botaniczna 3, 31-503 Krakow, Poland; 3 Laboratory of Drug Addiction Pharmacology, Institute of Pharmacology, Polish Academy of Sciences, Smętna 12, 31-343 Kraków, Poland

**Keywords:** Alzheimer’s disease, Amyotrophic lateral sclerosis, Antioxidant defense molecule, Non-enzymatic and enzymatic antioxidant, Oxidative stress biomarker, Parkinson’s disease, ESR—electron spin resonance, IST—immuno-spin trapping technique

## Abstract

The pathophysiologies of neurodegenerative diseases, including amyotrophic lateral sclerosis (ALS), Parkinson’s disease (PD), and Alzheimer’s disease (AD), are far from being fully explained. Oxidative stress (OS) has been proposed as one factor that plays a potential role in the pathogenesis of neurodegenerative disorders. Clinical and preclinical studies indicate that neurodegenerative diseases are characterized by higher levels of OS biomarkers and by lower levels of antioxidant defense biomarkers in the brain and peripheral tissues. In this article, we review the current knowledge regarding the involvement of OS in neurodegenerative diseases, based on clinical trials and animal studies. In addition, we analyze the effects of the drug-induced modulation of oxidative balance, and we explore pharmacotherapeutic strategies for OS reduction.

## Introduction

Identifying factors that contribute to neurodegenerative processes in the brain is one of the major goals of modern medicine. Currently, there are several hypotheses concerning the mechanisms that lead to the damage and death of brain cells in neurodegenerative diseases, such as excitotoxic effects by excitatory amino acids, disturbed cellular energy metabolism, and oxidative stress (OS), which is caused by free radicals or other reactive molecules.

The excessive production of reactive species and insufficient activity of antioxidant defense mechanisms have been implicated in the pathogenesis of many neurodegenerative diseases, including amyotrophic lateral sclerosis (ALS), Parkinson’s disease (PD), Alzheimer’s disease (AD), and Huntington disease (HD) [1].

## Reactive Species, Sources, and Cellular Pro-/Antioxidant Balance

It is thought that the biological oxidants that cause oxidative damage comprise the products of endogenous and exogenous processes that involve oxygen and nitrogen. Reactive species that contain oxygen are produced during aerobic respiration, cellular metabolism, and defense against pathogens [2]. The chemical potential of the oxygen molecule relies on its electron structure (two unpaired electrons in its basic triplet state). It promotes one-electron reactions that form the basis for respiration (reduction of oxygen molecules in four single-electron reactions), microsomal electron transport chains (ETC) (via cytochrome P-450 (CYP 450)), and oxidative burst activity in macrophages [3].

The high dynamics of the chemical processes that are achieved in elementary single-electron reactions are desirable and are the source of reactive molecules, which are either undesirable side products (respiration and metabolism) or in excess of the established requirements (defense process). These reactive molecules are known as reactive oxygen species (ROS) and reactive nitrogen species (RNS). Among them, the best known are singlet oxygen (^1^O_2_), superoxide anion radicals (O_2_^−•^), hydroxyl radicals (HO^•^), hydrogen peroxide (H_2_O_2_), nitric oxide (NO), and peroxynitrite anions (ONOO^−^) [4, 5].

At physiological concentrations, ROS/RNS play important regulatory and mediator functions, but an uncontrolled increase in ROS/RNS concentrations leads to a chain of radical reactions that increases the risk of damage to biological molecules in a living organism. This is caused by the high reactivity of ROS and RNS with lipids, proteins, carbohydrates, and nucleic acids. Hence, establishing an antioxidant barrier is required to limit the amount of ROS/RNS to a level that is not threatening to the integrity of biological systems. Excessive formation of ROS/RNS that exceeds the maximum capacity of the antioxidant barrier leads to a disturbance in the pro-/antioxidant equilibrium and, finally, to the development of the state known as OS.

**Fig. 1:**
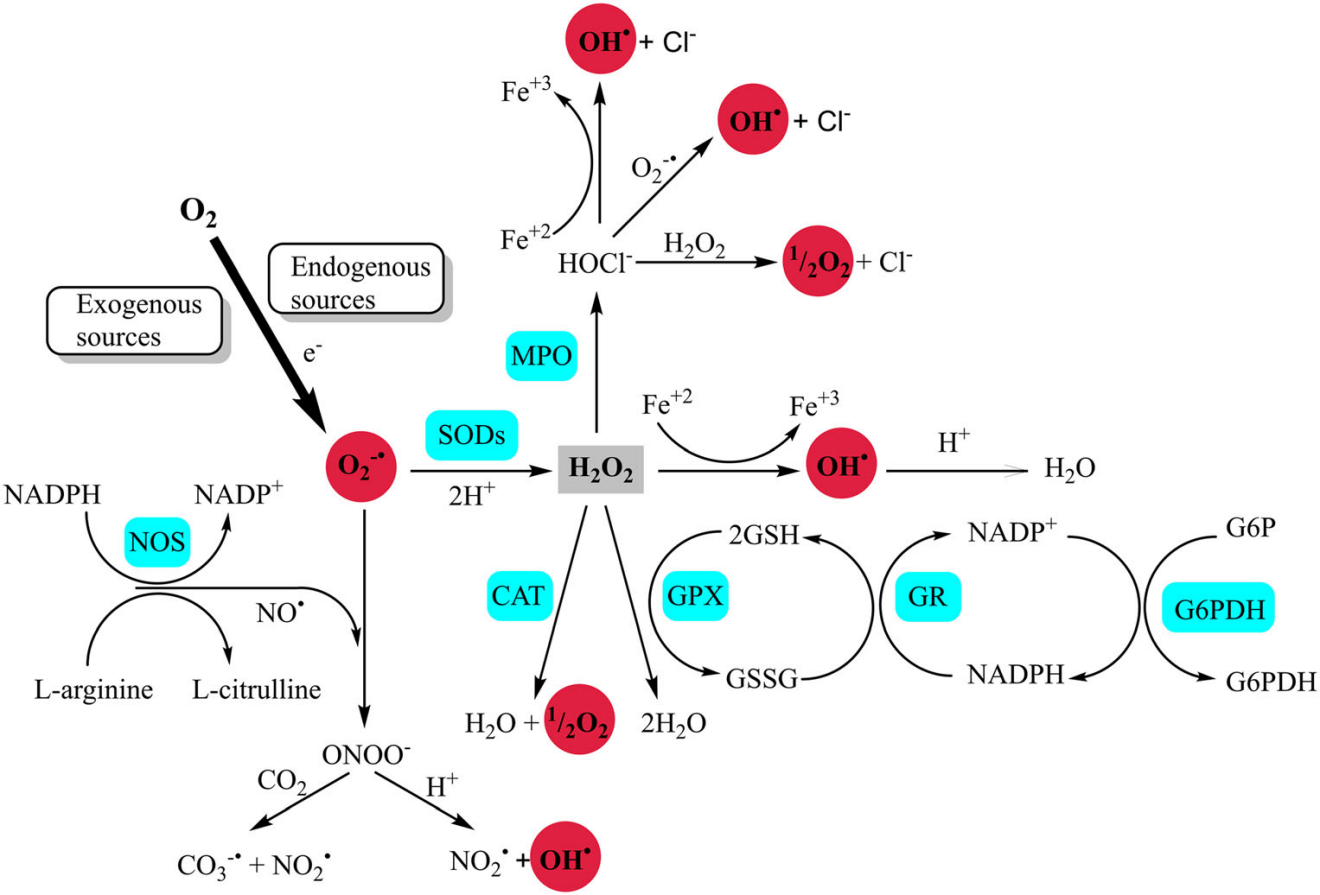
Generation of reactive species (based on [280–283])

OS can be triggered by radicals produced by either exogenous processes (e.g., xenobiotics, cold, viral and bacterial infections, ionizing radiation, ultrasound or photo-oxidation, poor diet, alcohol consumption, and smoking) or endogenous processes, which are the basic biochemical reactions in the body mentioned earlier (Fig. 1).

**Fig. 2:**
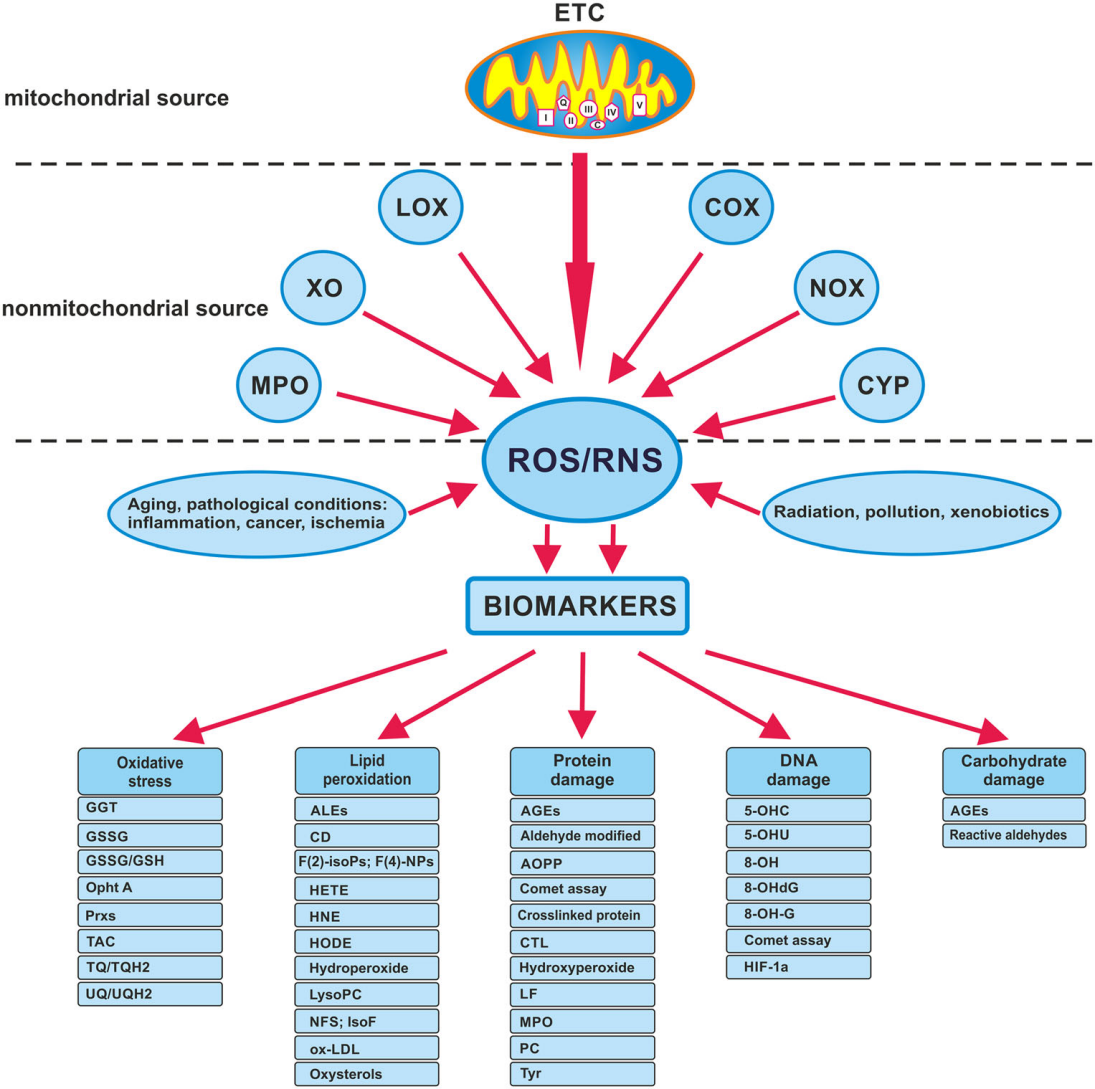
Source of radical and OS biomarkers. *8-OHA* 2,8-hydroxyadenine, *HNE* 4-hydroxynonenal, *5-OHC* 5-hydroxycytosine, *5-OHU* 5-hydroxyuracil, *8-OHdG* 8-hydroxy-2′-deoxyguanosine, *8-OH-Gua* 8-hydroxyguanine, *AGEs* advanced glycation end products, *ALES* advanced lipid peroxidation end products, *AOPP* advanced oxidation products, *CTL* creatol, *COX* cyclooxygenases, *CYP* cytochrome P450, *ETC* electron transport chain, *F2-isoPs* F2-isoprostanes, *F4-NPs* F 4-neuroprostanes, *HETE* hydroxyeicosatetraenoic acids, *HODE* hydroxyoctadecadienoic acid, *HIF-1a* hypoxia-inducible factor-1a, *IsoF* isofuran, *LF* lipofuscin, *LOX* lipoxygenases, *Lyso PC* lysophosphatidylcholines, *MPO* myeloperoxidase, *NOX* NADPH oxidases, *NFS* neurofuran, *Opht A* ophthalmic acid, *GSSG* oxidized glutathione, *ox-LDL* oxidized LDL, *GSSG/GSH* oxidized/reduced glutathione, *Prxs* peroxiredoxins, *PC* protein carbonyl, *TBARS* thiobarbituric acid reactive substances, *TQ/TQH2* tocopherylquinone/tocopheryl hydroquinone, *TAC* total antioxidant capacity, *Tyr* tyrosines, *UQ/UQH2* ubiquinone/ubiquinol, *XO* xanthine oxidase

ROS production as a side effect of aerobic respiration occurs on the inner membrane of the mitochondrion [6] (Fig. 2). The respiratory chain (mitochondrial ETC) consists of a series of membrane-bound complexes, such as complex I (reduced form of nicotinamide adenine dinucleotide (NADH)/ubiquinone reductase), complex II (succinate ubiquinone reductase), complex III (ubiquinol cytochrome c reductase), complex IV (cytochrome c oxidase), and complex V (adenosine triphosphate (ATP) synthase) [7]. The final acceptor of electrons and protons, an oxygen molecule, undergoes four-electron reduction, which can lead to the production of water molecules. During ETC, single electrons leak to reduce molecular oxygen and to form O_2_^−•^ and, later, H_2_O_2_ and HO^•^ [8] (Fig. 1).

Because of its high consumption of oxygen and its high lipid content, the brain is particularly vulnerable to damage caused by ROS and RNS. The extent of the damage varies, depending on, among other factors, the source and type of the reactive species. More active molecules, such as HO^•^ and ONOO^−^, interfere with other molecules at the site of their production, while less active ones, such as ^1^O_2_ and O_2_^−•^, can diffuse over longer distances and produce effects in more specific locations [9]. They can also react with each other and form more active forms, as in the case of the reaction of O_2_^−•^ with NO, which creates ONOO^−^ [10].

In mitochondria, NO is produced from l-arginine and l-citrulline in a reaction that is catalyzed by nitric oxide synthase (NOS), which has three isoforms with different tissue localizations. Cellular Ca^2+^-dependent neuronal NOS (nNOS) is expressed in astrocytes, microglia, and macrophages, and endothelial NOS (eNOS) is expressed in the vascular endothelium and Ca^2+^-independent inducible NOS (iNOS). NO is involved in many important processes within the central nervous system, such as the regulation of cerebral blood flow and memory. In addition, it plays a significant role in the regulation of the immune system, including the modulation of cytokine production. The released NO acts on neighboring cells, leading to somatic mutations and affecting cell cycle regulatory proteins, apoptosis, and DNA repair [11].

RNS are important for the generation of OS. ONOO^−^ is rapidly decomposed into HO^•^, nitrogen dioxide radical (NO_2_^•^), and nitryl cation (NO_2_^+^). All of these can damage nerve cells [12].

These highly reactive compounds induce changes in the structure and function of cell membranes, proteins, lipoproteins, enzymes, hormones, and genetic material. In particular, membranes are a primary target for ROS. Conversion products of lipid peroxidation lead to the decomposition of polyunsaturated fatty acids and the formation of the final products, i.e., the reactive aldehydes, such as malondialdehyde (MDA) and 4-hydroxynonenal (HNE). These compounds react with DNA or protein molecules and modify their structure and functions [13, 14].

There are several mechanisms designed to protect the organism from the harmful effects of ROS and RNS. The ultimate amount of ROS/RNS is under strict control in the body as a result of enzymatic and non-enzymatic defense mechanisms. The production of ROS- and RNS-induced damage (the final effect of OS) in tissue can be confirmed by the presence of tissue-specific and non-specific biomarkers [15–20]. Several markers of OS and antioxidant activity are presented in Fig. 2. Recent technical advances used to detect and identify ROS/RNS biomarkers and free radical metabolism are electron spin resonance (ESR), the immuno-spin trapping technique (IST), and radioimmunoassay (RIA) [21, 22].

The cellular antioxidant system, designed to prevent damage to tissue, is composed of antioxidant enzymes and other non-enzymatic compounds that have the ability to reduce different chemical structures [21]. These compounds are responsible for maintaining the balance between pro- and antioxidant agents and alleviating OS (see Table 1). The essential components of the enzymatic antioxidant defense are superoxide dismutase (SOD), catalase (CAT), glutathione peroxidase (GPx), and glutathione reductase (GR), while the non-enzymatic antioxidants include glutathione (GSH), thioredoxin (Trx), vitamins A, E, and C, flavonoids, trace elements, and proteins, e.g., albumin, ceruloplasmin, and metallothionein.

**Table 1:** Enzymatic and non-enzymatic antioxidants against OS

Agents	Mechanism
Enzymatic	
Superoxide dismutases (SOD) CuZn-SOD, located in the cytoplasm Mn-SOD, located in the mitochondria EC-SOD extracellular	Scavenges superoxide anion to form H_2_O_2_
Catalases (CATs)	Peroxisomes remove the hydrogen peroxide
Peroxiredoxins (Prxs)	Reduce free hydrogen peroxide to water
Glutathione peroxidases (GPx)	Catalyzes the reduction of H_2_O_2_ and alkyl hydroperoxides to H_2_O
Glutathione reductases (GRs)	Catalyzes the reduction of oxidized glutathione (GSSG) to reduced glutathione (GSH)
Glutathione *S*-transferases (GSTs)	Catalyze the conjugation of xenobiotics and endogenous or exogenous electrophilic compounds to reduced glutathione
Thioredoxin reductases (TrxRs)	Repair oxidative damages of proteins
Heme oxygenase-1 (HO-1)	Creation of products with antioxidant properties
Metalloproteins	
Metallothionein (MT)	Physiological metal chelation (Zn, Cu, Se) and xenobiotic metals (Cd, Hg, Ag, As)
Albumin	Chelation of metal ions (Fe, Cu)
Ceruloplasmin (CP)	Chelation of metal ions (Cu)
Ferritin	Chelation of metal ions (Fe)
Transferrin	Chelation of metal ions (Fe)
Non-enzymatic	
Vitamin C (ascorbic acid)	ROS scavenger
Vitamin E (α-tocopherol)	ROS scavenger
Vitamin A (retinol)	ROS scavenger
Carotenoids	ROS scavenger
Melatonin	ROS scavenger
Reduced nicotinamide adenine dinucleotide phosphate (NADPH)	Coenzyme used in anabolic reactions
Adenosine (ADO)	Endogenous activator of the cellular antioxidant defense system
Uric acid	ROS scavenger
Ubiquinol (the reduced form of coenzyme Q-10)	ROS scavenger
Polyphenols	ROS scavenger, metal chelation, induction of antioxidant enzymes
Phenolic acids	
Flavonoids	
Stilbenes	
GSH reduced glutathione	ROS scavenger
Amino acids (cysteine, homocysteine, methionine, taurine)	ROS scavenger
α-Lipoic acid	ROS scavenger
Lycopene	ROS scavenger
Carotenoids	ROS scavenger
S-adenosyl-L -methionine	ROS scavenger
Selenium	ROS scavenger

Data from [274–279]

A large body of evidence confirms a relationship between OS and the development of neurodegenerative diseases. The increased neuronal ROS production and accumulation of oxidative damage that occurs with age correlate well with the extent of neurodegeneration. In the following sections of this article, we present the current knowledge on the relationships between the intensity of OS and the initiation and progression of the major neurodegenerative diseases AD, PD, and ALS. The assessment of these relationships is based on biomarkers of OS and indicators of the pro-/antioxidant balance in nervous tissue that are correlated with the typical characteristics of neurodegenerative processes.

### Amyotrophic lateral sclerosis (ICD-10 G12.21)

ALS (also known also as Charcot’s or Lou Gehrig’s disease) is a severe neurodegenerative disease that is characterized by progressive upper motor neuron loss in the cerebral cortex and lower motor neuron loss in the brainstem and spinal cord [23, 24]. This results in spasticity, muscle wasting, and weakness, leading finally to paralysis and difficulties with speech, swallowing, and breathing. ALS may have either a limb onset (80 % cases) or a bulbar onset (20 % cases) [24, 25]. There is currently no cure for ALS and only riluzole, which acts on glutamate signaling, has been registered for the treatment of the disease. Riluzole was shown to slow disease progression and to improve limb function; however, the survival of patients was prolonged by only 2–3 months and death due to respiratory failure occurred in most cases within 3–5 years of the diagnosis [26].

The main pathological hallmark of ALS is the formation of cytoplasmic aggregates in degenerating motor neurons and surrounding oligodendrocytes, but those inclusions are also present in the frontal and temporal cortices, hippocampus, and cerebellum [27].

Only 5–10 % of ALS cases are inherited (familial ALS (FALS)); the remaining cases have no clear genetic background (sporadic ALS (SALS)) [24], and their pathogenesis is still poorly understood. Studies concerning the mechanisms of ALS development indicate that many factors, including excitotoxicity, mitochondrial dysfunction, endoplasmic reticulum stress, neuroinflammation, and OS, can be involved in this process. The two forms of the disease are clinically indistinguishable because the symptoms and pathological changes in SALS and FALS are similar. FALS is caused by mutations in some genes, such as those coding for SOD1, FUS RNA binding protein, TAR DNA binding protein, vesicle-associated membrane protein B, valosin-containing protein, optineurin, alsin, senataxin, spatascin, angiogenin, or ubiquilin-2 [24, 26]. Some of these gene mutations have also been found in SALS patients [28, 29].

The most common known genetic mutation for ALS is the recently described expanded GGGGCC hexanucleotide repeat in the non-coding region of the *C9Orf72* gene, on chromosome 9p21 [30]. Another common mutation is localized in *SOD1*. SOD1 in its native state metabolizes O_2_^−•^ to molecular misfolding [31]. Pathogenic mutations of SOD1 enzyme can be present in different regions of the enzyme, e.g., G93A (glycine 93 changed to alanine), H46R (histidine at codon 46 changed to arginine), or A4V (alanine at codon 4 changed to valine). Mutated SOD1 can form cytotoxic protein aggregates alone or with other proteins what possibly leads to loss of the enzymatic function or to acquiring the toxic properties [32, 33]. As SOD1 plays a crucial role in O_2_^−•^ clearance, its functional loss can lead to increased levels of OS. Then, the remaining wild-type SOD1 may become itself a target of oxidative modification after which it dissociates from dimers to monomers and further forms aggregates with toxic properties of mutant forms of SOD1. That was shown in in vitro studies [34, 35]. Accumulation of abnormal SOD1 was also confirmed in the spinal cord [34] in animal studies [36, 37] as well as in ALS patients [38].

## Clinical Studies

### OS Biomarkers

*Post*-*mortem* studies on tissue samples from SALS and FALS patients support the hypothesis of oxidative damage of proteins, lipids, and DNA. For instance, HNE and 3-nitrotyrosine (3-NT) levels were increased in CSF in SALS patients [39, 40], while a rise in 8-hydroxy-2′-deoxyguanosine (8-OHdG) has been described in both SALS and FALS patients [41, 42]. However, no correlation between these markers and the severity or duration of the disease has been found [43].

Raised levels of thiobarbituric acid reactive substances (TBARS) and advanced oxidation protein products (AOPP) and decreased ferric-reducing ability of plasma (FRAP) were detected in the plasma or erythrocytes of SALS patients [44–46], but plasma protein carbonyl (PC) levels surprisingly did not differ between SALS patients and control subjects [16]. More conclusive results came from a study where urine from SALS patients contained a higher level of isoprostanoids (IsoPs) and 8-OHdG compared to a control group [16, 47], suggesting that IsoPs and 8-OHdG could be considered markers of OS in ALS. These studies included only 50 participants with SALS compared to 46 control subjects. Thus, these results should be confirmed in larger cohorts.

The presence of OS biomarkers in regions of the CNS that are critical for ALS suggests that they are implicated in motor neuron degeneration. This fact is supported by very recent positron emission tomography (PET) imaging data in humans, which confirmed that OS were enhanced in the motor cortex in ALS patients compared with controls. Moreover, the observed OS increase in the mild stage of the disease led to the conclusion that OS may be an important factor associated with the development of neurodegeneration in ALS patients [48].

### Antioxidant Defense Biomarkers

Most studies concerning antioxidant defense biomarkers in ALS patients have shown changes in peripheral tissues or in CSF but rarely in the brain. For example, GSH levels were reduced in erythrocytes in ALS patients [45]. In contrast, an earlier study demonstrated a lower GSSG/GSH ratio with a concomitant rise in NO in the CSF of SALS patients [49]. According to the latter study, the lower GSSG/GSH level was caused by the decreased production of O_2_^−•^-oxidizing GSH because of the shift of oxidation to NO production or oxidation [49]. Very recent in vivo imaging studies have shown decreased GSH levels in the motor cortex of ALS patients by using the J-editing technique or ^62^copper-diacetyl-bis(N4-methylthiosemicarbazone (^62^CU-ATSM)) PET technique [25, 48].

The activity of SOD decreased in red blood cells and the CSF of FALS- and SALS-diagnosed patients [42, 50–52]. Interestingly, the reduction in the SOD1 protein concentration in erythrocytes of FALS patients correlated with *SOD1* gene mutations [42]. However, SALS patients displayed enhanced activity of SOD1 in CSF [53], indicating that this different outcome may depend on either the ALS type, the disease duration, or the sampling time [54].

Apart from SOD, the activity of CAT, another antioxidant defense enzyme, was also found to be diminished in red blood cells in FALS [51] and SALS [51, 52] patients. Another study revealed that CAT activity decreased in erythrocytes with disease progression, which may suggest a link between this parameter and ALS duration [45].

Results regarding GPx or GR activity in ALS patients are controversial. GPx type 3 (also known as plasma GPx) and GR levels were lower in the serum or in red blood cells, respectively, in ALS patients [45, 55]. In contrast, another study showed enhanced GPx in the serum and CSF of ALS patients [56], while GR activity was elevated in the erythrocytes in both SALS and FALS patients [51, 52]. One study reported no change in GR activity in red blood cells in SALS patients [57].

In ALS patients, the plasma and/or CSF levels of other antioxidants (i.e., vitamin E, β-carotene, vitamin C, uric acid, and thiol group-containing molecules, such as ubiquinol-10) were either elevated [53] or not changed [44, 58, 59].

These divergent results could be explained by the heterogeneity of ALS [60]. In fact, ALS-diagnosed patients vary in their rate of disease progression and may differ in the number of years of survival. The median survival from onset to death in ALS varies from 20 to 48 months; however, many studies have reported a survival time of longer than 10 years in 10–20 % of patients [60]. Moreover, a study from Cova et al. [57] showed that the activity of GPx, GR, and CuZn SOD in erythrocytes was decreased in patients who had a faster disease progression rate and that SOD1 activity significantly decreased along the disease course in subjects with a more impaired functional status. All of these results support the hypothesis that ALS has many variants or mimic syndromes that differ in their pathogenic mechanisms and in their profile of enzymatic and non-enzymatic antioxidant responses [57].

### Pharmacological Strategies to Reduce OS

Several pharmacotherapeutic agents with antioxidant properties have been attempted to slow ALS progression; however, most of them failed to do so (Table 2). Vitamin E, when combined with riluzole, diminished TBARS levels, enhanced GPx activity in the plasma, and prolonged the milder stages of the ALS Health State scale, but it did not affect survival and motor function [61].

**Table 2:** Clinical trials in ALS patients with agents/drugs showing antioxidant properties

Antioxidant	Time point of antioxidant status determination from the beginning of therapy	Oxidative damage biomarker change	Reference
Vitamin E (500 mg twice a day) with riluzole (100 mg/day)	3 months	↑GPx in plasma, ↓TBARS in plasma	[61]
Selegiline hydrochloride (5 mg twice a day p.o.)	6 months	nd	[69]
Acetylcysteine (50 mg/kg/day s.c.)	12 months	nd	[68]
Creatine (5 g twice a day p.o.)	16 months	nd	[67]
Coenzyme Q10 (1,800 and 2,700 mg/day p.o.)	9 months	nd	[66]
Edaravone (60 mg/day i.v.)	2 weeks administration every 2 weeks, 6 replications	↓3-NT in CSF	[64]
Melatonin (300 mg/day) per rectum	4 months	↓PC in serum	[70]

↓ decrease, ↑ increase, *nd* not determined, *3*-*NT* 3-nitrotyrosine, *8*-*OHG* 8-hydroxyguanosine, *CSF* cerebrospinal fluid, *GPx* glutathione peroxidase, *i.v*. intravenously, *p.o*. per os, *PC* protein carbonyl, *s.c*. subcutaneous, *TBARS* thiobarbituric acid reactive substances

Another study investigated a very high dose of vitamin E as an add-on therapy to riluzole for ALS treatment; however, this treatment with vitamin E, at a dose of 5,000 mg/day for 18 months, failed to slow disease progression [62]. On the other hand, another study showed that intake of a combination of polyunsaturated fatty acids and vitamin E reduced the risk of developing ALS [63].

Edavarone (MCI-186) is another free radical scavenger that is already approved to treat cerebral infarction and to investigate ALS. It eliminates lipid peroxide and hydroxyl radicals by transferring an electron to the radical and thereby exerting a protective effect on neurons. In ALS patients, edavarone was shown to diminish nitrosative stress (NS) in CSF [64] and is now being tested in a phase III clinical trial in Japan for ALS treatment [65]. Coenzyme Q10 was administered for 9 months in a dose of 2,700 mg/day to ALS patients in a multicenter trial that unfortunately showed a lack of compound efficacy for ALS treatment [66]. This study also determined 8-OHdG levels in the plasma; however, according to the authors, the results for this measure will only be available later [66].

Other antioxidant medications, i.e., acetylcysteine, creatine, or selegiline, had no significant effect on survival or the rate of clinical progression of ALS [67–69]. In these studies, the influence on antioxidant defense biomarkers and oxidant damage was not evaluated. In another study, melatonin was used as a potential neuroprotective compound and, when normalized to control values, was found to elevate the level of PC in the serum of 31 SALS patients [70].

The novel antioxidant AEOL 10150, which is a small molecule that catalytically consumes ROS and RNS, is considered to be the most promising compound under evaluation in a clinical trial [71]. In a small, open-label study in ALS patients, AEOL 10150 was shown to be a safe and well-tolerated drug [71]. However, neither efficacy nor measurement of oxidative biomarkers in ALS patients who were on AEOL 10150 has been published.

The antioxidant medications used in ALS clinical trials have so far been unable to slow the progression of the disease. Moreover, a limited number of clinical studies have investigated oxidative damage or changes in the antioxidant defense status after antioxidant therapy.

### Anti-ALS Drugs that Modulate the Oxidative Balance

No data.

## Animal Studies

### OS Biomarkers

The most popular animal models of ALS are based on SOD1 mutant transgenic mice (for example: SOD1 G93A, SOD1 H46R, SOD1 H46R/H48Q, SOD1 A4V, etc. (for more details, see [72])). Other ALS models include Wobbler mice and rodent models with genetic mutations in TAR DNA binding protein of 43 kDa (TDP-43), VAMP-associated protein B, dynactin or FUS/TLS, or C9Orf72 [73].

Changes in oxidative biomarkers have been detected in ALS models in rodents (Table 3). For example, free radical levels were elevated in the spinal cord of SOD1 G93A mice *post-mortem* [74, 75], and trapped radical adducts were also recently detected in the spinal cord of these mice in vivo [76]. In line with this latter observation, increased levels of PC in the spinal cord and in the motor cortex [77, 78], MDA and 4-HDA in the brain and CSF [79], and HNE, HNE-adducts, and 8-OHdG in the spinal cord [80, 81] have been observed in several ALS animal models (see Table 3).

**Table 3:** OS biomarkers in ALS animal model

Animal model	OS biomarkers	Reference
Mice SOD1 G93A-2Gur	↑PC in spinal cord	[77]
Mice SOD1 G93A	↑MDA, ↑4-HDA in brain and CSF Ø MDA, Ø 4-HDA in skeletal muscles and heart Ø PC in brain, CSF, skeletal muscles, liver, heart	[79]
Mice SOD1 G93A	↑MDA, ↑HNE in spinal cord	[81]
Mice SOD1 G93A	↑In vivo trapped radical adducts	[76]
Mice SOD1 G93A	↑8-OHdG in spinal cord	[80]
Mice SOD1 G93A	↑PC and ↑3-NT in motor and sensory CTX	[78]
Rats SOD1 G93A	↑Overnitrated proteins in mononuclear cells from peripheral blood	[83]
Mice SOD1 G93A	↑3-NT in spinal cord in presymptomatic stage	[82]

↓ decrease, ↑ increase, *Ø* no changes, *nd* not determined, *3*-*NT* 3-nitrotyrosine, *4*-*HDA* 4-hydroxyalkenal, *8*-*OHdG* 8-hydroxy-2′-deoxyguanosine, *CSF* cerebrospinal fluid, *CTX* cortex, *MDA* malondialdehyde, *PC* protein carbonyl

It has been established that enhanced nitration processes are also present in animal models of ALS. For example, 3-NT was found in the spinal cords of aged SOD1 G93A mice together with the presence of overnitrated proteins (actin or ATPase) in the spinal cord [82], in the motor and sensory cortices [78], and in the peripheral blood mononuclear cells [83] in transgenic mouse models of ALS and that these were observed even before the onset of the disease.

OS and NS are widely present in ALS, and in vitro studies suggest that this mechanism can facilitate the formation of protein aggregates [84]. In support of this conclusion, recent studies have revealed that a selective nNOS inhibitor increased survival in SOD1 transgenic mice [85].

### Antioxidant Defense Biomarkers

In general, transgenic mouse models overexpressing mutant human *SOD1* showed increased activity by the enzyme [32]. However, in the end stage of the disease, SOD1 activity in SOD1 G93A mice remained at the same level as wild-type SOD in non-transgenic mice [86]. What is interesting is that SOD1 knockout mice did not show an ALS-like phenotype [32].

Other antioxidant biomarkers, including GSH, GPx, and GR, are also changed during the course of ALS. The levels of GSH differed in mice carrying different SOD1 mutations. For example, in SOD1 G93A mutant mice, the GSH level decreased and the level of GSSG rose in the lumbar spinal cord [87], while in the same tissue in SOD1 mice with the H46R/H48Q mutations, the GSH level was not changed [88]. A recent study by Vargas et al. [88] showed that decreased GSH content accelerated neurological deficits in the SOD1 G93A mouse model. The mechanism of GSH reduction is linked to the nuclear transcription factor Nrf2 because its transfection into the SOD G93A mouse brain led to the up-regulation of GSH synthesis in astrocytes and reduced the most apparent neurological and biochemical symptoms of the disease [89].

In animal models of ALS, motor neurons have displayed overexpression of Prx2 and glutathione peroxidase-1 (GPx1). The number of neurons containing Prx2 and GPx1 decreased in the terminal stage of ALS [90], suggesting a breakdown of this redox system at the advanced stages of the disease. As discussed by Kato et al. [91], a possible reason for Prx2 and GPx1 breakdown might be related to the co-aggregation of these enzymes with mutant SOD1 and the exacerbation of mutant SOD1-mediated toxicity in neuronal Lewy body-like hyaline inclusions (LBHIs), which was shown in the spinal cords of transgenic rats [91]. Similarly, GPx3 protein levels in the serum of SOD1 H46R rats were increased in the pre-symptomatic stage and decreased gradually with disease progression [55]. However, another study did not reveal significance for the role of GPx in ALS [92], and further investigation is therefore necessary to clarify this problem.

Because enhanced SOD activity in ALS animal models remains enhanced in most of the disease stages and decreases only to the control level of non-transgenic mice in the end stage of the disease, this raises the question of whether these ALS animal models are suitable to study ALS antioxidant defense biomarkers because an ALS key enzyme, SOD, is decreased in ALS patients. These contradictory results, obtained in animals and humans, do not allow researchers to draw conclusions regarding the significance of these biomarkers in animal ALS models.

### Pharmacotherapeutic Strategy to Reduce OS

Many substances possessing antioxidant properties have been proposed as ALS treatment strategies (Table 4). For example, coenzyme Q10 was shown recently to be unable to prolong survival when given after ALS onset [93]. As coenzyme Q10 is characterized by rather poor CNS availability (which possibly explains its small pharmacological effects), its reduced form, ubiquinol-10, has better bioavailability and antioxidant properties and was also investigated. However, similar to its parent drug, ubiquinol-10 did not prolong lifespan. Nevertheless, it was noted that poor CNS availability after oral dosing was observed in this study, which possibly explains the lack of pharmacological effects, similar to the case with its parent drug [93].

**Table 4:** Therapeutic trials with agents/drugs with antioxidant properties in ALS animal models

Drug, dose, route of administration	Animal model	Therapy start after	Changes in oxidative defense and damage biomarkers	Onset change	Survival change	Reference
Ubiquinol-10 800 mg/kg/day p.o.	Mice SOD1 G93A	Post-onset	nd	*Ø*	*Ø*	[93]
Coenzyme Q10 800 mg/kg/day p.o.	Mice SOD1 G93A	Post-onset	nd	*Ø*	*Ø*	
Creatine (2 % in diet)	Mice SOD1 G93A	Pre-onset	nd	*Ø*	↑	[95]
Creatine (2 % in diet) + celecoxib (12 % in diet)	Mice SOD1 G93A	Pre-onset	nd	*Ø*	↑	[94]
Creatine (2 % in diet) + rofecoxib (5 % in diet)	Mice SOD1 G93A	Pre-onset	nd	*Ø*	↑	
Creatine (2 % in diet) + minocycline (22 mg/kg/day i.p.)	Mice SOD1 G93A	Pre-onset	nd	*Ø*	↑	[96]
Vitamin E 200 IU/day, 5 days/week for 30 days	Mice SOD1 G93A	Pre-onset	↓8-OHG in spinal cord	↑	ø	[103]
EUK-8 and EUK-134 33 mg/kg i.p. 3 times a week	Mice SOD1 G93A	Pre-onset	↓MDA, ↓PC in spinal cord	*Ø*	↑	[102]
Edaravone 15 mg/kg i.p.	Mice SOD1 G93A	At symptoms onset	↓abnormal SOD1 deposition in spinal cord	na	*Ø*	[36]
AEOL 10150 2.5 mg/kg i.p.	Mice SOD1 G93A	At symptoms onset	↓MDA, ↓3-NT in spinal cord	na	↑	[108]
Ammonium tetrathiomolybdate 5 mg/kg i.p.	Mice SOD1 G93A	Pre-onset	↓LPO, ↓SOD in spinal cord	↑	↑	[86]
		Post-onset	nd	↑	↑	[105]
Resveratrol 25 mg/kg i.p.	Mice SOD1 G93A	Pre-onset	↓MDA in spinal cord	↑	↑	[106]
Melatonin 30 mg/kg, 10 μl/g body weight i.p.	Mice SOD1 G93A	Pre-onset	nd	↑	↑	[104]
Melatonin 5 mg/kg, 2.5 mg/kg or 50 mg/kg i.p.	Mice SOD1 G93A	Pre-onset	↑4-HNE, ↑SOD1 in spinal cord	↓	↓	[107]

↑ increase, ↓ decrease, *0* no change, *na* not applicable, *nd* not determined, *3*-*NT*- *3* nitrotyrosine, *i.p*. intraperitoneally, *LPO* lipid hydroperoxide, *MDA* malondialdehyde, *PC* protein carbonyls, *ROS* reactive oxygen species, *s.c*. subcutaneously, *SOD* superoxide dismutase

Creatine was the next drug that extended survival, but not age of onset, in ALS animals [94–96]. It has neuroprotective properties and buffers against ATP depletion in mitochondria. Its dysfunction can lead to ATP decreases, which may contribute to cell death [97]. Because mitochondrial swelling and vacuolization are among the earliest pathological features in ALS mice with the SOD mutations [98], creatine could be useful for protecting mitochondria and for disease treatment in such a preclinical model. In most studies, creatine was delivered in the diet as a 1 or 2 % food supplementation. In addition to its effect of prolonging the lifespan in a mouse ALS model, creatine also improved motor performance [96] and provided protection from motor neuron loss at 120 days of age in these mice [98]. Moreover, creatine was shown to prevent the rise of 3-NT in the spinal cord and ROS production in the dialysate from microdialysis in ALS animals [98], and it also had a positive effect on weight retention [99]. However, one study found that creatine administration had no effect on the disturbed muscle function [100]. Moreover, co-administration of creatine with the cyclooxygenase-2 inhibitors rofecoxib and celecoxib or of creatine with minocycline (a tetracycline antibiotic with neuroprotective and anti-inflammatory effects) [101] may be even more beneficial for extending survival (even by 30 %) [94, 96].

Other antioxidants that extended survival in ALS mice include EUK-8 and EUK-134. In preclinical studies, these two synthetic SOD/CAT mimetics reduced the levels of OS, as measured by MDA, PC, and prolonged survival, but not disease onset, in ALS mice [102]. On the other hand, treatment with vitamin E significantly delayed ALS onset with no effect on survival but with a diminishing effect on 8-hydroxyguanine (8-OHG) levels in the spinal cord [103]. Drugs that shared both effects (ALS onset delay and lifespan extension) were melatonin, ammonium tetrathiomolybdate (a copper-chelating drug), and resveratrol (a substance that originates in plants and is found in highest amounts in red wine and the skin of red grapes) [86, 104–106]. However, in case of melatonin, the data were not clear as it was given in a dose range of 2.5–50 mg/kg intraperitoneal (i.p.) (in the same animal model) and also produced a surprisingly shortened survival, accelerated disease onset, enhanced lipid peroxidation in the spinal cord, and increased spinal motoneuron loss [107]. A possible reason for these results might be due, according to authors, to melatonin’s effect on upregulating the gene expression of human toxic SOD1, which presumably overrides any of the anti-oxidant properties of melatonin. Such a hypothesis needs to be tested in future studies.

Other substances used in preclinical ALS models, i.e., resveratrol, which, when given before disease onset, decreased MDA levels [106], and ammonium tetrathiomolybdate, which decreased spinal SOD activity [86]. The latter compound also ameliorated ALS-like symptoms in SOD1 G93A mice, probably by chelating the copper ions from the Cys111 site in the SOD-1 enzyme, which is important because various SOD-1 mutations affect Cu and Zn metal-binding, thereby promoting toxic protein aggregation [86]. Moreover, successful effects were achieved from treatment with ammonium tetrathiomolybdate when it was given after disease onset, which is satisfactory because this regime of administration closely reflects clinical practice.

Edaravone and AEOL 10150 are recently studied drugs for ALS that were administered at ALS onset. Although edaravone did not prolong survival in an ALS mouse model, it diminished SOD1 deposition in the anterior horn of the spinal cord and slowed disease progression and motor neuron degeneration [36]. Another substance that gave more promising results is AEOL 10150, which, when administered to ALS mice, decreased 3-NT and MDA levels in the spinal cord, extended animal survival [108], provided better preservation of motor neuron architecture, and diminished the level of astrogliosis [108].

In conclusion, according to animal studies, antioxidants may become putative ALS therapies because many of them extend the lifespan and diminish OS in ALS animals. However, it must be remembered that many of them, when tested in humans, have not yet shown benefits for survival time and motor function amelioration.

### Anti-ALS Drugs that Modulate Oxidative Balance

Riluzole was shown to extend the lifespan in mice in the SOD1 G93A ALS animal model, but it did not change the disease onset [109] or have a satisfactory effect on the latter parameter [110]. No studies concerning oxidative defense or oxidative damage biomarkers were conducted; however, in vitro studies showed that riluzole had antioxidant properties in cultured cortical neurons [111].

### Parkinson’s Disease (ICD-10 G20)

PD is a progressive degenerative disorder that is characterized by the loss of dopamine-producing neurons in the substantia nigra (SN) and by the presence of Lewy bodies in the SN and locus coeruleus. Clinical manifestations of PD include resting tremor, muscle rigidity, slowing of voluntary movements (bradykinesia), a tendency to fall (postural instability), and a mask-like facial expression [112]. The pathological hallmarks of PD, Lewy bodies, contain various proteins, including α-synuclein, ubiquitin, Parkin, and neurofilaments.

PD occurs in sporadic (non-inherited; 90–95 % cases) and familial (inherited; 5–10 % cases) forms. Mutations in the α-synuclein gene cause one of the familial forms of PD via autosomal dominant inheritance [113]. Recently, it was suggested that OS and imbalances between ROS/RNS and antioxidant defense mechanisms are linked to the etiology of PD.

## Clinical Studies

### Oxidative Biomarkers

Many studies have demonstrated the presence of OS and its markers in the brain and CSF in PD patients. Cholesterol lipid hydroperoxide and MDA were found to be up to 10-fold higher in SN in *post-mortem* brains of PD patients compared with other brain regions and age-matched controls [114]. In contrast, a recent paper demonstrated lower levels of MDA in the caudate nucleus and putamen and increased MDA levels in the frontal cortex in the *post-mortem* analyses of PD brains compared to healthy age-matched controls. Those results suggested that the non-SN regions, such as the caudate nucleus or the putamen, may have different compensatory mechanisms against OS could protect them from oxidative damage [115]. Moreover, HNE and acrolein-modified proteins were found in the neocortex and brainstem and in the SN, respectively, of PD patients [116–118]. According to in vitro studies, HNE modification of α-synuclein has been shown to trigger oligomerization and fibrillization of unmodified α-synuclein in the nervous system, which might lead to dopaminergic neuron injury. A recent observation suggests that HNE-modified proteins should be considered to be important players in PD pathophysiology [119].

Despite a number of studies supporting lipid peroxidation in the brains of PD patients, the levels of F2-isoprostanes were not elevated in the SN of PD patients [120], and the reason for this observed difference needs to be explained.

PCs were also found in PD patients’ brains compared to age-matched controls [121]. PCs are present not only in the brain regions specific for PD (the SN, caudate nucleus, and putamen) but also in other brain areas not directly linked with PD. Because most PD patients are treated with l-DOPA, this observation may suggest that l-DOPA, the “gold standard” for PD therapy, may contribute to carbonyl formation because it was shown to have pro-oxidant properties in vitro [122]. Interestingly, brain regions from individuals with putative presymptomatic PD (incidental Lewy body disease) showed no PC rise. This information suggests that in PD, oxidative protein damage occurs late during disease progression and/or that l-DOPA treatment contributes to carbonyl formation [121]. Oxidative damage to proteins in PD also occurred through nitration, and 3-NT was found to be increased within Lewy bodies in the SN pars compacta (SNpc) of PD patients [123].

Another pathology found in PD brains is DNA and RNA damage [124, 125]. The factors 8-OHG and 8-OHdG were elevated in various parts of the PD brain compared to controls; however, the most striking rise was detected in the SN [124, 125]. Similarly, increased levels of 8-OHG and 8-OHdG were observed in CSF [126, 127]. However, these studies came to opposite conclusions with respect for the correlation between 8-OHG levels and disease duration.

In the CSF of living PD patients, enhanced levels of HNE and MDA have been shown as well [128–130], but different results were obtained by Shukla et al. [131]. Moreover, markers of oxidative damage in PD patients were also detected in the serum and urine [132–135], but their use as indicators of the course of the disease is far from being useful for clinical practice because the existing data are contradictory [136–138]. As has been suggested [136–138], these differences may be due to the variability in methods used to measure OS markers.

The results of many studies have demonstrated the presence of OS in the brain, CSF, serum, and urine of PD patients; however, none of the OS markers has been established as a specific biomarker for PD disease or as a marker for PD disease progression.

### Antioxidant Defense Biomarkers

PD is characterized by a selective loss of GSH in the SN (40 % compared to control subjects) but not in other parts of the brain [139]. This decrease is one of the earliest biochemical changes that has been observed in the disease [140–143], and it results in a selective drop in mitochondrial complex I activity, another hallmark of PD [144]. On the other hand, a substantial rise in SOD levels has been observed in the SN and basal ganglia in PD patients [145], while no change in activities of CAT, GPx, and GR was found compared to age-matched controls [145]. Another study showed some deficiency in GPx in the SN in Parkinsonian patients [146], but the weak (ca. 19 %) reduction in such a small number of patients (*n* = 11) cannot be considered to be conclusive. Another small pilot study in PD patients (*n* = 8) indicated a peripheral (in blood) increase in GSH/GSSG [147] when the patients went off of PD medications (dopamine receptor agonists) for 12 h in comparison to GSH/GSSG levels when they were on medications. This suggests that PD medications may play a disadvantageous role that leads to enhanced peripheral oxidative stress; however, the small sample size excludes a final conclusion [147].

### Pharmacological Strategies to Reduce OS

Because there is an overwhelming amount of evidence showing that OS and NS in PD patients leads to an increase in pathological damage in the SN, many approaches have been attempted to reduce ROS/RNS production. One of the possible strategies was to supplement GSH. As shown by Sechi et al. [148], in nine patients in the early stage of the disease [148], GSH (600 mg twice daily) administered intravenously for 30 days reduced (by 42 %) the clinical disability of PD patients, and this effect lasted for 2–4 months. Furthermore, a recent study of *N*-acetylcysteine (150 mg/kg), which is a GSH precursor, revealed an elevation in brain GSH levels and the blood GSH/GSSG ratio after 1-h of intravenous (i.v.) infusion [149]. Unfortunately, no results concerning the clinical status of PD patients have been described. However, *N*-acetylcysteine, in a smaller dose (70 mg/kg p.o., every 12 h over 2 days), produced negligible changes in GSH levels in the CSF and no immediate improvement to symptoms (according to the Unified Parkinson’s Disease Rating Scale and the Montreal Cognitive Assessment) [150].

Magnetic resonance imaging (MRI) studies showed a rise in iron concentrations in the SN in PD patients [151]. Because iron can lead to ROS production in PD patients, an iron-binding compound, deferiprone, has been tested in a pilot study in PD patients (FAIRPARK trial, registered as ClinicalTrials.gov NCT00943748). Patients in early-stage PD who were on a stabilized dopamine regimen received deferiprone (30 mg/kg/day) for 12 months (early-start paradigm, *n* = 19), while the other group received the drug for only 6 months, with the latter, shorter therapy starting 6 months later than the early-start paradigm (delayed-start paradigm, *n* = 18) [152]. The earlier therapy start diminished SN iron deposits to a greater extent than the delayed-start paradigm and improved motor performance vs. placebo and vs. the delayed-start paradigm, according to the Unified Parkinson’s Disease Rating Scale [152]. Moreover, in deferiprone-treated patients, GPx and SOD activity in the CSF increased, which supports the connection between the chelator treatment and the antioxidant response.

Vitamin E (α-tocopherol) was also suggested as a way to diminish the OS and to reduce clinical symptoms in PD. However, the clinical trial The Deprenyl and Tocopherol Antioxidant Therapy of Parkinsonism study (DATATOP study) revealed no evidence of any beneficial effect for α-tocopherol (2,000 IU/day) in either slowing functional declines or ameliorating the clinical features of Parkinson’s disease. It is of note that no analysis of OS biomarkers was performed in that trial [153, 154]. Since DATATOP, no clinical trials using vitamin E as a potential PD medication have been conducted. In fact, vitamin E was only used in PD clinical trials as a supplement for coenzyme Q10 or as a placebo [155] or a control [156].

Another potent antioxidant, coenzyme Q10 (1200 mg a day), in the first reported multicenter, randomized, placebo-controlled, and double-blind trial slowed functional declines compared to placebo [157]. Lower doses or different formulations of coenzyme Q10 displayed no symptomatic effects on midstage PD [158]. Recent clinical trial did not show a benefit for coenzyme Q10 (1,200 or 2,400 mg/day) in 600 patients in early-stage PD [155].

An early clinical study on creatine monohydrate (10 g/day for 12 months) in 67 subjects who were within 5 years of a PD diagnosis showed some positive results for treating behavioral difficulties [159], and an additional 18-month follow-up study confirmed this finding [160]. On the other hand, a smaller (*n* = 31 creatine PD group, *n* = 17 placebo PD group), but longer, 2-year clinical trial demonstrated that creatine had no effect on PD scores or dopamine transporter imaging [161]. In September 2013, the NINDS discontinued the NET-PD LS-1 study (phase III clinical trial with a total of 1,720 planned participants, ClinicalTrials.gov identifier: NCT00449865) that started in 2007 because the results obtained from a study of creatine used for the treatment of early stage PD did not demonstrate a statistically significant difference between the active substance and placebo [162].

In conclusion, although evidence for the link between OS and damage in PD is overwhelming, suggesting the potential efficacy of antioxidant drugs, most clinical trials have so far failed to support this statement.

### Anti-Parkinsonian Strategies to Restore Oxidative Balance

Administration of zonisamide, an anticonvulsant drug prescribed to treat resting tremor in PD, inhibited the rise of 8-OHdG levels in the urine of PD patients. As the 8-OHdG rise correlates with disease progression and aging, it can be presumed that zonisamide could be helpful in defending against OS-evoked DNA modifications in PD patients. Other drugs used for treatment of PD (i.e., l-DOPA, monoamine oxidase B (MAO-B) inhibitors, catechol-*O*-methyltransferase (COMT) inhibitors, and the dopamine receptor agonists ropinirole and pramipexole) have not shown such properties [163].

Interesting findings were reported in a study that measured GSH levels in venous blood in PD subjects who were on- and off-medication while performing acute physical exercises, because we know that this type of physical activity leads to GSH depletion and GSSG rise [164]. Surprisingly, the off-medication patients had a lower drop in GSH level than the on-medication group. This finding suggests that patients in the off-medication state handled acute stress better than those in the on-medication state, indicating that medication may impede the ability to tolerate acute OS [165]. Similar conclusions were obtained in a very recent study by Nikolova et al. [166], who observed a greater rise in PC and 8-OHdG levels in the blood of PD patients who were treated with l-DOPA in comparison to untreated PD patients, demonstrating that administration of l-DOPA may cause greater OS than PD itself [166].

## Animal Studies

### Oxidative Biomarkers

The most popular animal models of PD include pharmacological (6-hydroxydopamine (6-OHDA), 1-methyl-4-phenyl-1,2,3,6-tetrahydropyridine (MPTP), rotenone, and paraquat) as well as several genetic (with mutations in the α-synuclein, PINK1, Parkin, or LRRK2 genes) models [167].

The 6-OHDA model (Table 5), wherein the toxin is injected directly into the SNpc, medial forebrain bundle, or striatum, was the first animal model of PD associated with dopaminergic neuronal death within the SNpc [168].

**Table 5:** Changes in OS and anti-OS defense biomarkers in toxin-based model of PD

Animal model	Route of administration, dosage	Animal species	OS biomarkers	Oxidative defense biomarkers	Reference
6-OHDA	Unilateral intra-STR inj. 10 μg/2 μl	Rats (Wistar)	↑TBARS in SN	↓GSH, ↓CAT ↓SOD in SN	[191]
	Bilateral intra-STR inj. 10 μg/2 μl	Rats (Fischer)	↑HNE, ↑PC in STR	nd	[176]
	Unilateral intra-STR inj. 300 μg/10 μl	Rats (Sprague-Dawley)	↑PC in STR	↓GSH and ↓SOD in STR	[177]
	Intra-STR inj. 8 mg/2 ml	Rats (Wistar)	↑MDA in STR	↓SOD, GST in STR	[178]
	Intra-STR inj. 8 μg/4 μl	Rats (Wistar)	↑3-NT, ↑HNE in STR	nd	[179]
MPTP	20 μl/kg intra-SN inj.	Rats (Wistar)	↑MDA in SN	↓SOD in SN	[180]
	25 mg/kg/day for 5 days	Mice (C57BL/6)	↑iNOS, ↑HNE, ↑3-NT in SN	nd	[181]
	Intra-SN inj.	Rats (Wistar)	↑MDA, ↑nitrate in STR and CTX	↓GSSH, ↓CAT in STR and CTX	[182]
	24 mg/kg sc. for 6 days	Mice (C57BL/6)	↑MDA in STR	↓GSH, ↓SOD, ↓GPx, ↓CAT in STR	[197]
	100 μg/1 μl, bilateral infusions	Rats (Wistar)	↑LPO in STR	↓GSH, ↑SOD in STR	[183]
	15 mg/kg s.c. for 3 days	Mice (C57BL/6)	↑3-NT, ↑HNE in ventral midbrain	↓GSH, ↑GSSG in ventral midbrain	[187]
	24 mg/kg s.c. (2 times/day at 12-h interval) for 2 days	Mice (C57BL/6)	↑8-OHG in SN	nd	[188]
	20 mg/kg i.p. 3 times at 2-h interval	Mice (C57BL/6J)	↑8-OHG in STR	nd	[189]
	30 μg/kg i.p. 2 times at 16-h interval	Mice (Balb/c)	nd	↓GSH, ↑SOD in SN, nucleus caudate, and putamen	[192]
Rotenone	2.5 mg/kg, i.p., for 10 days	Rats (Wistar)	nd	↓GSH in HIP, ↓CAT in HIP and STR	[193]
	One-sided intra-SN inj. 6 μg	Rats (Sprague-Dawley)	nd	↓GSH, ↑Cu-Zn SOD, ↑CAT in SN, Ø GSH, Ø SOD in STR	[194]
	3 mg/kg i.p. for 30 days	Rats (Wistar)	nd	↓GSH, ↓SOD in STR	[195]
	2 mg/kg s.c. for 35 days	Rats (Sprague-Dawley)	↑TBARS, ↑SAG in the midbrain regions and cerebellum and CTX	↓GSH, ↓CAT in midbrain	[196]
	2 mg/kg s.c. for 35 days	Rats (Sprague-Dawley)	↑MDA in midbrain regions and cerebellum	↓GSH, ↓SOD, and ↑CAT in midbrain and CTX ↑NO in midbrain	[184]
	1 mg/kg i.p for 3 weeks	Mice (CFT-Swiss)	↑PC in HIP, CTX, STR	↓GSH, ↓TT in HIP, ↓GPx, ↓CAT in CTX and HIP, ↓SOD in CTX and STR	[186]
	1.5 mg/kg/48 h s.c., 6 doses	Rats (Albino)	↑LPO, ↑PC, ↓mtDNA in STR	↓GSH, ↓SOD in STR	[185]
Paraquat + maneb	Paraquat (10 mg/kg) + maneb (30 mg/kg), twice a week, for 9 weeks	Mice (Swiss albino)	↑MDA, ↑NO in nigrostriatal tissues	↑GST in nigrostriatal regions	[190]
	Paraquat (10 mg/kg, i.p.), and maneb (30 mg/kg, i.p.), twice a week, for 6 weeks	Mice (C57BL6/J)	α-Synuclein radical in the midbrain	nd	[175]

↑ increase, ↓ decrease, *Ø* no change, *nd* not determined, *6-OHDA* 6-hydroxydopamine, *CAT* catalase, *CTX* cortex, *GPx* glutathione peroxidase, *GSH* glutathione, *GST* glutathione *S*-transferase, *HIP* hippocampus, *HNE* 4-hydroxynonenal, *i.c.v*. intracerebroventricular, *intra*-*SN inj*. intra-substantia nigra injection, *LPO* lipid hydroperoxide, *MDA* malonyldialdehyde, *MPTP* 1-methyl-4-phenyl-1,2,3,6-tetrahydropyridine, *NO* nitric oxide, *NOS* nitric oxide synthetase, *PC* protein carbonyl, *SAG* superoxide anion generation, *s.c*. subcutaneous, *SN* substantia nigra, *SOD* superoxide dismutase, *STR* striatum, *TBARS* thiobarbituric acid reactive substances

Another PD model utilizes MPTP, a highly lipophilic molecule that rapidly crosses the blood–brain barrier, leading to an irreversible and selective loss of dopaminergic neurons in the SN in non-human primates [169, 170] and in rodents [162, 171], although the latter species was less sensitive to MPTP than primates [172]. Other chemical models are based on an insecticide, rotenone, or paraquat, an herbicide. Rotenone, when given i.v. in a low dose to rats, produces selective degeneration of SN dopaminergic neurons that is accompanied by α synuclein-positive LB-like inclusions [173]. Paraquat is used less widely than MPTP, rotenone, or 6-OHDA models and is used instead as an addition to other toxic agents, such as the fungicide maneb [174]. It was reported to cause selective degeneration of nigrostriatal dopaminergic neurons in mice [175]. The mechanism of action of MPTP (a precursor form of the reactive metabolite MPP+), rotenone, or paraquat is based on the inhibition of mitochondrial complex I, which leads to ROS production [167, 174, 175].

As has been demonstrated in numerous studies, OS is widely present in all of these toxin-based models (see Table 5). 6-OHDA, when injected into the striatum, increased MDA, HNE, PC, and 3-NT levels in this structure and increased TBARS in SN [176–179]. Similarly, in the MPTP and in the rotenone models, elevated levels of lipid peroxidation products [180–185] and oxidatively modified proteins [185, 186] were observed in various parts of the brain (striatum, cortex, SN, hippocampus, cerebellum, and midbrain). In addition to lipid damage, increased 3-NT levels were also detected following the use of MPTP in the SN, striatum, and ventral midbrain [181, 187]. MPTP or rotenone-treated animals also showed oxidatively modified RNA or DNA in the SN or striatum [185, 188, 189]. In the paraquat and maneb PD models, enhanced lipid peroxidation in the nigrostriatal areas of animal brains was also shown [190] (Table 5). A very recent report from Kumar et al. [175] demonstrated for the first time that α-synuclein radical is formed in the midbrain in paraquat- and maneb-treated mice, indicating that radical formation is mediated by peroxynitrite and depends on both NADPH oxidase and iNOS. This interesting result regarding α-synuclein radical formation was obtained by using the immuno-spin trapping method in combination with immunoprecipitation [175]. Moreover, it was noted that protein radicals such as α-synuclein radical may trigger protein aggregation, which plays a causal role in dopaminergic neuronal death [175].

For review of genetic models and OS, see the excellent paper [167].

All toxin-based models share common characteristics, including the ability to produce ROS and further oxidative damage, which causes death in dopaminergic neurons and reflects part of the pathology observed in PD. Although all of those models have drawbacks, they are useful for testing neuroprotective therapies.

### Antioxidant Defense Biomarkers

A characteristic shared feature observed in all toxin-based models is a drop in GSH level in key PD structures [177, 183–187, 191–196] (Table 5). Importantly, lower GSH levels make nigrostriatal neurons more susceptible to oxidative damage and further degeneration.

Studies using 6-OHDA also showed a reduction in activity by SOD, CAT, and glutathione *S*-transferase (GST) in striatum and SN [177, 178, 191]. On the other hand, results from the MPTP model are inconclusive regarding SOD and CAT activity. Moreover, some of the MPTP studies showed increased SOD activity in the SN [192] and striatum [183], while others reported diminished SOD activity in these regions [180, 197]. These differences may have resulted from the use of different doses of the toxin, varied routes of drug administration (intracranial versus i.p. or s.c.), and different use of rodent species in the studies (see Table 5).

Similar to SOD activity, CAT activity cannot be considered a biomarker of OS in rodent PD models as its activity was both diminished [182, 191, 193, 194, 196, 197] and enhanced [184]. Moreover, GPx activity was diminished in striatum in an MPTP model [197], while GST activity was found to be elevated in a maneb and paraquat PD animal model [190] (Table 5).

All of these reports on the enhanced activities of SOD, CAT, and GST suggest the presence of mechanisms in brain areas that defend against exposure to PD toxin models. On the other hand, diminished activities or levels of antioxidant enzymes may indicate that these defense mechanisms were overcome and that the degeneration process had begun.

### Pharmacotherapeutic Strategy to Reduce OS

Several agents, such as valproic acid [178] and melatonin [198], effectively reversed changes in antioxidant defense biomarkers and oxidative damage in the 6-OHDA rat model of PD (Table 6).

**Table 6:** Therapeutic trials with substances possessing antioxidant properties in PD animal models and their influence on changes of biomarkers of OS and anti-oxidative defense

Model	Biomarkers of OS and anti-oxidative defense	Drug and route of administration	Changes in biomarkers of OS and anti-oxidative defense	Reference
6-OHDA, rats (Wistar)	↑MDA, ↓SOD, ↓GPx, ↓CAT in STR	Melatonin (10 mg/kg i.p. for 30 days)	↓MDA, ↑SOD, ↑GPx, ↑CAT in STR	[198]
6-OHDA, 8 mg/2 ml intra-STR inj., rats	↑MDA, ↓SOD, ↓GST in STR	Valproic acid (300 mg/kg) i.p. for 10 days	↓MDA, ↑SOD in STR	[178]
MTPT intra-SN inj., rats	↓GSH, ↓CAT, ↑nitrate, ↑LPO in STR and CTX	Ceftriaxone (100 mg/kg or 200 mg/kg i.p.)	↑GSH, ↑CAT, ↓nitrate, ↓LPO in STR and CTX	[182]
		Ceftriaxone (100 mg/kg i.p. and ropinirol 1, 5 or 3 mg/kg i.p.)	↑GSH, ↑CAT, ↓nitrate, ↓LPO in STR and	
MPTP mice (C57BL/6J),	↑LPO, ↑SOD, ↓GPx, ↓GSH in SNpc	*N*-acetylcysteine	↓LPO, ↓SOD, ↑GPx, ↑GSH in SNpc	[199]
Rotenone, 2.5 mg/kg, i.p. for 10 days, rats	↓GSH in HIP, ↓CAT in HIP and STR	Ibuprofen (15 mg/kg, p.o. 22 days post 10-day rotenone treatment)	↑GSH in HIP vs. rotenone group, ↑CAT in HIP and STR vs. rotenone group	[193]
Rotenone, 3 mg/kg i.p. for 30 days, rats	↓GSH, ↓SOD, ↑MDA in STR	Lycopene (10 mg/kg, p.o. for 30 days, a combined treatment with rotenone)	↑GSH, ↓SOD, ↑CAT in HIP, STR	[195]
Rotenone, 1 mg/kg/day i.p. for 3 weeks, mice (CFT-Swiss)	↓GSH in HIP, ↓TT in HIP, ↓GPx, ↓CAT in CTX and HIP, ↓SOD in CTX and STR, ↑PC in HIP, CTX, STR	TSE—aqueous extract of tomato seeds (100 mg/kg p.o. for 3 weeks, 1 h before rotenone injection)	↑GSH, ↑CAT, ↑TT in HIP, ↓PC in STR, ↑SOD in CTX and STR, ↑GPx in CTX and HIP ↓PC in STR, ↑SOD in CTX and STR	[186]
Rotenone, 1.5 mg/kg/48 h/6 doses, s.c., rats (Albino)	↑LPO, ↑PC, ↓GSH, SOD, ↓mtDNA in STR	Acetyl-l-carnitine (100 mg/kg/day, p.o.), α-lipoic acid (50 mg/kg/day, p.o.) or their combination	↓LPO, ↑GSH, ↑SOD, and ↑mtDNA in STR after each drug alone or after combination, ↓PC, ↑CAT in STR only after combination	[185]
Rotenone, 2 mg/kg s.c. for 35 days, rats (Spraque- Dawley)	↑MDA in midbrain and cerebellum, ↓GSH, ↓SOD, and ↑CAT in midbrain and CTX, ↑NO in midbrain	Centrophenoxine (100 mg/kg i.p., co-administration with rotenone for 35 days)	↓MDA in midbrain and cerebellum, ↑GSH, ↑SOD,↑CAT in midbrain and CTX,↓NO in midbrain	[184]
Maneb (30 mg/kg) and paraquat (10 mg/kg) i.p. twice a week for 9 weeks, mice (Swiss albino)	↑MDA, ↑NO, ↑GST in nigrostriatal regions	Silymarin (40 mg/kg i.p. for 9 weeks), maneb, and paraquat were administered 2 h after this injection	↓MDA, ↓NO, and ↓GST in nigrostriatal tissues	[190]
		Melatonin (30 mg/kg i.p. for 9 weeks, maneb and paraquat were administered 2 h after this injection		

↑ increase, ↓ decrease, *nd* not determined, *CAT* catalase, *CTX* cortex, *GPx* glutathione peroxidase, *GSH* glutathione, *GST* glutathione transferase, *GST* glutathione *S*-transferase, *HIP* hippocampus, *HNE* 4-hydroxynonenal, *i.c.v*. intracerebroventricular, *i.p*. intraperitoneal, *intra-SNc inj.* intra-substantia nigra injection, *intra*-*STR inj*. intrastriatal injection, *LPO* lipid hydroperoxide, *MDA* malonyldialdehyde, *NO* nitric oxide, *NOS* nitric oxide synthetase, *PC* protein carbonyl, *s.c*. subcutaneous, *SN* substantia nigra, *SNpc* substantia nigra pars compacta, *SOD* superoxide dismutase, *STR* striatum, *TBARS* thiobarbituric acid reactive substances, *TT* total thiol

There are also data in the literature showing that other agents and drugs have antioxidant activity (i.e., ceftriaxone [182] and *N*-acetylcysteine [199], which decreased oxidative damage and/or enhanced antioxidant defense biomarkers in the striatum, cortex or SN in rodent MPTP models).

Ibuprofen (a non-steroidal anti-inflammatory drug [193]), acetyl-l-carnitine (a natural compound reported to prevent mitochondrial injury deriving from oxidative damage in vivo), α-lipoic acid (given alone or in combination with acetyl-l-carnitine [185]), and centrophenoxine (a potent nootropic agent that acts as an antioxidant) [184] were demonstrated to enhance GSH levels and CAT and SOD activity and to decrease lipid peroxidation in investigated brain regions in a rat rotenone model (Table 6).

Prevention of oxidative damage and the presence of antioxidant defense biomarkers have been documented following treatment with natural compounds, such as lycopene [195], aqueous extract of tomato seeds (TSE) [186], and melatonin [190].

Many different agents may improve antioxidant brain status in different PD models. However, it should be noted that most of these agents were given before or concomitantly with rotenone, MPTP, or other PD-causing toxins. To definitively answer whether these agents can also show efficacy in reducing the consequences of exposure to prior administration of PD-inducing toxins, further studies are required. This is especially true because the latter type of drug administration would be a better model for evaluating any pharmacological strategy for reducing OS in PD patients.

### Anti-Parkinsonian Drugs that Modulate Oxidative Balance

Most anti-parkinsonian drugs may improve brain antioxidant status in PD preclinical tests (Table 7). Ropinirole, a second-generation, non-ergoline dopamine receptor agonist with D2-like receptor selectivity and a chemical structure similar to that of dopamine was found to enhance GSH levels and CAT [182] activity and to diminish nitrate levels [182] in the striatum in MPTP-lesioned animals.

**Table 7:** Therapeutic trials with anti-parkinsonian drugs in PD animal models and their influence on biomarkers of OS and of anti-oxidative defense

Model	Oxidative defense biomarkers	Drug	Changes in oxidative defense biomarkers	Reference
MPTP 25 mg/kg i.p. for 5 days administered to 6C57BL/6J mouse	↓GSH in SN	l-DOPA (200 mg/kg i.p. 2 injections/day for 4 weeks, co-administration with MPTP	Ø GSH in SN	[200]
MPTP 1 μmol/2 μl intra-SN administered to Wistar rat	↓GSH, ↓CAT, ↑nitrate, and ↑LPO in STR and CTX	Ropinirole 1, 5, or 3 mg/kg i.p. for 14 days, after MPTP treatment	↑GSH, ↑CAT, ↓nitrate (only 1.5 mg/kg) in STR and CTX	[182]
MPTP 25 mg/kg i.p. for 5 days administered to C57BL/6J mouse	↓GSH in SN	Pramipexole 1 mg/kg i.p. 2 injections/day for 4 weeks, co-administration with MPTP	↑GSH in SN	[200]
Rotenone 2 mg/kg s.c. 35 days administered to Sprague-Dawley rats	↑TBARS, ↑SAG in midbrain regions and cerebellum, ↓GSH and ↓CAT in midbrain regions and CTX	Deprenyl 10 mg/kg p.o. co-administration with rotenone for 35 days	↓TBARS, ↓SAG in midbrain; ↑GSH, ↑CAT in midbrain and CTX	[196]
6-OHDA 300 μg/10 μl unilateral intra-STR inj. administered to Sprague-Dawley rats	↑PC, ↓GSH, and ↓SOD in STR	Deferoxamine 50 mg/kg p.o. for 14 days, co-administration with 6-OHDA	↓PC, ↑GSH, and ↑SOD in STR	[177]

↑ increase, ↓ decrease, *Ø* no change, *na* not applicable, *6-OHDA* 6-hydroxydopamine, *CAT* catalase, *CTX* cortex, *GSH* glutathione, *i.p*. intraperitoneal, *LPO* lipid hydroperoxide, *MPTP* 1-methyl-4-phenyl-1,2,3,6-tetrahydropyridine, *PC* protein carbonyl, *s.c*. subcutaneous, *SAG* superoxide anion generation, *SOD* superoxide dismutase, *STR* striatum, *TBARS* thiobarbituric acid reactive substances

Other anti-parkinsonian drugs, such as selegiline (a selective irreversible MAO-B inhibitor) [196], deferoxamine [177], and pramipexole (a non-ergoline dopamine agonist) [200], increased GSH levels in the striatum, SN, or cortex. Deferoxamine also decreased a protein oxidative damage biomarker [177] and enhanced SOD activity in the striatum, while selegiline reduced superoxide anion generation (SAG) and increased CAT activity in midbrain regions and the cortex [196]. Interestingly, l-DOPA, the most commonly used drug in PD treatment, did not restore the reduced GSH levels in the SN in the MPTP mouse model [200].

The above studies suggest that antiparkinsonian drugs, with the exception of l-DOPA, display some antioxidant properties, which may be considered as part of their mode of action and efficacy in PD treatment.

### Alzheimer’s Disease (ICD-10 G30)

AD is the most common neurodegenerative disease and is characterized by memory loss, dysfunctions in cognitive abilities (e.g., executive function, attention, language, and visuospatial skills), confusion, aggression, and mood swings [113, 201] and leads to death within 5 to 9 years after the diagnosis [202].

The pathogenesis of AD is not yet clearly understood. The aggregation of extracellular insoluble protein plaques (composed of beta amyloid (Aβ)) and intracellular neurofibrillary tangles (NFTs, composed of tau protein) are critical hallmarks of AD [113, 201]. However, many ongoing pathological processes lead to regional neuron loss, beginning in the medial temporal lobe [201] and following in other brain regions, such as the hippocampus and cerebral cortex [113].

Many clinical trials and animal studies have recognized free radicals as mediators of injury in AD patients and AD models.

## Clinical Studies

### Oxidative Biomarkers

The first report of the involvement of OS in AD pathology came from a paper by Martins et al. [203], in which elevated activities of glucose-6-phosphate dehydrogenase and 6-phosphogluconate dehydrogenase were documented in *post-mortem* studies of brains of AD patients compared to age-matched controls [203]. The latter increase was proposed to be a response to enhanced brain peroxide metabolism.

Other *post-mortem* studies on brains and CSF from AD patients showed ROS-mediated injuries. For instance, AD patients had increased levels of MDA and HNE, iso- and neuroprostanes, and acrolein compared to controls [204]. It was suggested that these peroxidated lipids formed adducts with proteins and that they might thereby play a role in AD pathogenesis [201].

In addition to lipids, protein damage due to OS has also been reported in AD. In fact, increased PC levels in the frontal and parietal cortices and the hippocampus were found in *post-mortem* studies of the brains of AD patients, while PC was absent in the cerebellum, where no AD pathology was present [205]. Furthermore, evidence of oxidative DNA modification was found in AD patients as an increase in 8-OHG in human brain homogenates [201].

In AD patients, ROS production seems to be enhanced; furthermore, increases in RNS were also detected. Such evidence of RNS modification was identified both in astrocytes and in neurons in AD patient brains examined *post-mortem* [206]. The changes in astrocytes were found to co-localize with an increase in iNOS, eNOS, and nNOS expression. The latter increases were noted specifically in cortical pyramidal cells [206]. In another study, increased expression of iNOS and eNOS was observed to be directly associated with Aβ deposits, showing that beta amyloid might induce NOS to produce NO, which might lead to 3-NT formation [207].

The presence of 3-NT was also reported in the cerebral blood vessels of AD patients *post-mortem* [206]. These findings were associated with reduced NO bioavailability in plasma and further hypoperfusion in AD patients because NO promotes vascular smooth muscle relaxation and thereby regulates blood flow. As was reported in recent studies, enhanced NO production is a likely cause of production of ONOO^−^ [208, 209].

Another set of oxidative damage biomarkers, 8-OHdG and 8-OHG, were elevated in AD ventricular CSF [210] and in brains in both mitochondrial and nuclear DNA compared with age-matched controls [211].

Consistent data showing enhanced levels of MDA, HNE, iso- and neuroprostanes, acrolein, PC, 8-OHG, 8-OHdG, and 3-NT in the CNS of AD patients can be considered to be proof that OS and NOS are significant contributors to brain damage.

### Antioxidant Defense Biomarkers

Pivotal antioxidant enzymes, including GPx, CAT, and SOD, display changed levels in the brains of AD patients [212, 213]. However, the data are not consistent. For instance, elevated levels of antioxidant enzymes (mainly SOD) in the hippocampus and amygdala of AD patients have been reported [208]. On the other hand, in AD patients, decreased levels of SOD, GPx, and CAT were found in the frontal and temporal cortex [214], while decreases in GSH were observed in the brain and erythrocytes of AD patients [212, 213]. Evidence in support of changes in antioxidant enzymes comes from a recent study that identified genetic polymorphisms in the *GPx*-*1* and *GST* genes that were positive risk factors for AD [215, 216].

The GSH levels were reduced not only in AD but also in mild cognitive impairment (MCI), which is considered to be a preclinical stage of AD [217]. MCI patients also showed a decreased GSH/GSSG ratio and a reduction in SOD and GST activity in the hippocampus compared to age-matched controls [218], which suggests that alterations in GSH metabolism may be considered as an early biomarker of AD onset.

The plasma levels of antioxidants, such as albumin, bilirubin, uric acid, lycopene, vitamin A, vitamin C, and vitamin E, are decreased in AD patients [219, 220], although there are some reports indicating the opposite direction of these changes [221]. Differences in results might be caused by measurement of antioxidants at different disease stages (fully developed disease vs. subclinical stage of the disease) [219–221].

### Pharmacological Strategy to Reduce OS

As OS is present in AD patients, some clinical studies have aimed to test the ability of antioxidant substances to diminish ROS production and to alleviate or to slow the course of the disorder (Table 8).

**Table 8:** Clinical trials with substances/drugs with antioxidant properties in AD patients

Antioxidant	Duration of therapy	Oxidative damage biomarker change	Reference
Vitamin E (α-tocopherol, 800 IU/day) + vitamin C (500 mg/day) + α-lipoic acid (900 mg/day)	16 weeks	↓F2-isoprostane in CSF	[223]
Coenzyme Q10 (400 mg × 3 times/day)	16 weeks	Ø F2-isoprostane in CSF	[223]
ω-3 (3 g/day contained 675 mg DHA and 975 mg EPA)	12 months	Ø F2-isoprostane in urine, Ø PC in plasma	[228]
ω-3 + α-lipoic acid (ω-3, 3 g/day contained 675 mg DHA and 975 mg EPA + α-lipoic acid, 600 mg/day in one tablet)	12 months	Ø F2-isoprostane in urine Ø PC in plasma	[228]
Vitamin C (1,000 mg/day) + vitamin E (400 IU/day)	12 months	↓oxidation of CSF	[222]
Curcumin (1 or 4 g/day)	6 months	Ø F2-isoprostane in plasma	[230]
Curcuminoids (2 or 4 g/day)	24 weeks	Ø F2-isoprostane in CSF	[229]
Idebenone (120, 240, or 360 mg/day)	12 months	nd	[225]

↓ decrease, *Ø* no change, *nd* not determined, *CSF* cerebrospinal fluid, *DHE* docosahexaenoic acid, *EPA* eicosapentaenoic acid, *ω-3* omega-3 fatty acids

Most studies on the effects of the administration of vitamins that possess antioxidant activity have provided inconclusive information showing that they diminished lipid peroxidation in CSF but had no positive effects on cognitive or functional aspects. For example, AD patients taking cholinesterase inhibitors and vitamin C (1,000 mg/day) or vitamin E (400 IU/day) supplements for 1 year showed decreased CSF oxidation, but no difference in cognition was observed [222]. Similarly, 16-week treatment with a combination of vitamin E (800 IU/day), vitamin C (500 mg/day) and α-lipoic acid (900 mg/day) decreased CSF F2-isoprostane levels, which suggested a reduction of OS in the brain, but surprisingly, this therapy accelerated cognitive decline (according to Mini-Mental State Examination scores), leading to the conclusion that a combination of antioxidants should not be used for AD therapy [223]. On the other hand, very recent results from a large, clinical, double-blind, randomized trial (TEAM-AD VA, NCT00235716) showed that a much larger dose of vitamin E (2,000 IU/day) than was used in previous trials resulted in a slower functional decline compared with placebo in mild to moderate AD [224]. Although the latter study suggests that vitamin E can have a positive influence on AD, no OS biomarkers have been measured in parallel in the AD patients who participated in that trial, which limits the final conclusion.

Administration of other antioxidants, including coenzyme Q10 as well as its synthetic analogue, idebenone (which possesses a better ability to pass the blood–brain barrier), in AD patients did not provide any positive results with regards to the volume of ROS-dependent tissue damage or cognitive function improvements [223, 225]. Similarly, administration of omega-3 (ω-3) fatty acids also did not yield a positive outcome for slowing the rate of decline of cognitive (Mental State Examination (MMSE); Alzheimer’s Disease Assessment Scale—cognitive subscale) or functional (Activities of Daily Living/Instrumental Activities of Daily Living) abilities [226, 227]. Different results were reported in a recent study, where 12-month ω-3 fatty acid supplementation caused a delay in progression of functional impairment in AD patients, while combined supplementation of ω-3 and α-lipoic acid resulted in slowing global cognitive declines (MMSE) [228]. Although positive cognitive outcomes were obtained, no changes after ω-3 or ω-3 plus α-lipoic acid supplementation were observed in OS biomarkers, suggesting a different mechanism for their actions that lead to improved cognitive and functional measures [228].

Curcumin, which is a natural polyphenolic compound and an in vitro blocker of Aβ aggregation, did not diminish the enhancement of F2-isoprostane levels in the CSF [229] or plasma [230], or the Aβ_1–40_ level in plasma [230], and it did not ameliorate neuropsychological test results in AD patients [229, 230]. As suggested by Ringman et al. [229], low bioavailability of the drug and low plasma levels due to poor uptake from the gastrointestinal tract might be the reasons for its lack of efficacy in the latter studies. There is some hope that curcumin efficacy can be improved through the use of its lipidated forms, which are predicted to have better uptake compared to the nonlipidated form [231]. In a study in healthy, middle-aged volunteers, the lipidated form of curcumin (80 mg/day) decreased Aβ_1–40_ levels in plasma [231], suggesting that further trials using lipidated curcumin should be considered in AD patients.

More promising results came from a study using resveratrol. The Copenhagen City Heart Study reported that monthly or weekly consumption of red wine was associated with a lower risk of dementia [232]. The clinical study NCT01504854, also called the “Phase II Study to Evaluate the Impact on Biomarkers of Resveratrol Treatment in Patients with Mild to Moderate AD,” was undertaken in 2011 to evaluate the effectiveness of resveratrol for changing AD CSF biomarkers (total tau, Aβ_42_, Aβ_40_, and phospho-tau181), the effect of resveratrol treatment on hippocampal atrophy and regional cortical thinning, and the influence of resveratrol administration on the outcomes of several clinical scales used to assess the severity of dementia [233]. According to the report at http://clinicaltrials.gov record (accessed 15 May 2015), the study has been completed, but no results have yet been published.

### Anti-Alzheimer’s Disease Therapy and Oxidative Balance

Acetylcholinesterase (AChE) inhibitors (donepezil, rivastigmine, galantamine, and tacrine) and the NMDA receptor antagonist memantine are the most commonly used drugs in AD pharmacotherapy. Only some clinical studies that have investigated the influence of these drugs on oxidative balance in AD patients are currently available (see Table 9). One of them showed no positive effects of AChE inhibitors on OS parameters (CAT and GR levels) in the blood of AD patients compared with AD drug-naïve patients [234]. In another study, donepezil enhanced GSH levels, while rivastigmine diminished advanced glycation end products (AGEs) in the plasma of AD patients. However, other examined parameters, namely total antioxidant capacity (TAC) and PC, have not been improved by those drugs [235]. Combined therapy with memantine and donepezil failed to improve GSH, TAC, PC, or AGEs [235]. A very recent study revealed that 6-month treatment with memantine decreased the oxidation rate of plasma lipids in AD patients compared with untreated patients [236]. The above clinical trials included small sample sizes and should initiate future examinations evaluating the effect of different types of AD medications on OS markers in AD patients.

**Table 9:** Clinical trials of anti-Alzheimer drugs and their influence on OS biomarkers

Anti-Alzheimer medication (dose)	Duration of therapy	Oxidative damage biomarker change	Reference
Donepezil (10 mg/day)	≥24 months	↑GSH, Ø AGEs, TAC, PC in plasma	[235]
Tivastigmine (9.5 mg/day)	≥24 months	↓AGEs, Ø GSH, TAC, PC in plasma	
Donepezil (10 mg/day) + memantine (20 mg/kg)	≥24 months	Ø GSH, TAC, PC, AGEs in plasma	
Memantine (20 mg/day)	For 6 months	↓oxidation rates of lipids in plasma	[236]

↑ increase, ↓ decrease, *Ø* no change, *AGEs* advanced glycation end products, *GSH* glutathione, *PC* protein carbonyl, *TAC* total antioxidant capacity

## Animal Studies

### Oxidative Biomarkers

AD can be modeled by several procedures in animal. Injection with scopolamine (i.p.), streptozotocin (intracerebroventricular (i.c.v.)), Aβ (i.c.v.), or apolipoprotein E (APOE) in transgenic models is used to study sporadic AD, while amyloid precursor protein (APP) and presenilin 1 (PSEN1) and PSEN2 transgenic models are used to examine familial AD [237–240]. For detailed descriptions of AD animal models, see [241–246].

In both pharmacological and genetic models of AD, disordered OS biomarkers are present in animal brains (Table 10). MDA, HNE, or TBARS were enhanced in all of those models in the cerebral cortex or/and hippocampus or/and the whole brain [237–241, 247–255]. Oxidative modification of proteins has also been demonstrated in the cortex and whole brain homogenate of transgenic AD mice [253, 255] and in the cerebral cortex and hippocampus of an Aβ_1-42_ mouse model [247]. In addition to OS due to oxygen, there is also proof of the presence of NS in whole brain lysates from the APP23 transgenic AD mouse model [251].

**Table 10:** OS biomarkers and OS defense biomarkers in pharmacologically developed and in transgenic AD animal models

Animal model (dosage)	Animal species	OS biomarkers	OS defense biomarkers	Reference
Aβ_1-42_ (i.c.v. injection)	Mice	↑MDA in cerebral CTX and HIP	↓SOD, ↓GPx, ↓GSH, ↑GSSG in cerebral CTX and HIP	[237]
Aβ_1-42_ injection (i.c.v. injection)	Mice (Chinese Kun Ming)	↑MDA in cerebral CTX and HIP	↓SOD, ↓GPx, ↓GSH in HIP and cerebral CTX	[241]
Aβ_1-42_ (i.c.v. injection 400 pmol)	Mice (C57BL/6)	↑MDA, ↑PC in cerebral CTX and HIP	↑Mn-SOD, ↑Zn, Cu-SOD, ↑GPx (only up to 2 days after Aβ_1-42_ injection), ↑GR (only 2 h after Aβ_1-42_ injection) in cerebral CTX and HIP	[247]
Scopolamine (2 mg/kg i.p. once per day for 2 weeks)	Mice (Kun Ming)	↑MDA in HIP	↓SOD, ↓GSH in HIP	[238]
Scopolamine (1 mg/kg, i.p. single injection)	Mice (Swiss)	↑MDA in CTX and HIP	↓SOD, ↓GPx, ↓GSH-Rx in CTX and HIP	[248]
Scopolamine (1.4 mg/kg, i.p. single injection)	Mice (Swiss)	↑MDA in whole brain lysate	↓CAT in whole brain lysate	[249]
AF64A, a cholinotoxin (2 nmol/2 μl, bilaterally single i.c.v. injection)	Rat (Wistar)	↑MDA in HIP	nd	[239]
Streptozotocin (3 mg/kg bilaterally i.c.v. injection on days 1 and 3)	Rat (Wistar)	↑MDA in whole brain lysate	↓GSH, Ø CAT, Ø SOD in whole brain lysate	[240]
Streptozotocin (2.57 mg/kg bilaterally single i.c.v. injection)	Mice (Swiss albino)	↑TBARS in HIP	↓GSH, ↓GPx, ↓GR in HIP	[250]
Streptozotocin (3.0 mg/kg i.c.v. single injection, 5 μl/injection per site)	Rat (Wistar)	↑HNE, ↑MDA, ↑TBARS, ↑PC in HIP	↓GSH, ↓GPx, ↓GR, ↓CAT, ↓SOD in HIP	[256]
AbPP Tg2576 transgenic mice		↑HNE, ↑3-NT in whole brain	nd	[251]
APP/PSEN1 transgenic mice		↑MDA in HIP	nd	[252]
APP23 transgenic mice		↑PC in CTX	nd	[253]
Heterozygote APP transgenic mice		↑TBARS in the brain homogenate	↓GSH, ↓SOD in the brain homogenate	[254]
APPswe/PS1dE9 transgenic mice		↑MDA, ↑PC in the brain homogenate	↓SOD, ↓GPx in the brain homogenate	[255]

↑ increase, ↓ decrease, *nd* not determined, *3*-*NT* 3-nitrotyrosine, *CTX* cortex, *GSH* glutathione, *CAT* catalase, *GPx* glutathione peroxidase, *GR* glutathione reductase, *GSSG* oxidized glutathione, *HIP* hippocampus, *HNE* 4-hydroxynonenal, *i.c.v.* intracerebroventricular, *i.p*. intraperitoneal, *MDA* malonyldialdehyde, *Mn*-*SOD* manganese superoxide dismutase (located in mitochondria), *PC* protein carbonyl, *SOD* superoxide dismutase, *TBARS* thiobarbituric acid reactive substances, *Zn*, *Cu*-*SOD* copper/zinc superoxide dismutase (located in cytoplasm)

### Antioxidant Defense Biomarkers

Antioxidant defense biomarkers have been found to be changed in AD models (see Table 10). Diminished levels of GSH in the cerebral cortex or hippocampus or in whole brain lysates have been demonstrated in pharmacologically induced AD animal models [237, 238, 240, 241, 247, 250, 254, 256]. Furthermore, the activities of enzymes connected with GSH metabolism, such as GPx and GR, and the enzymes involved in antioxidant defense (SOD and CAT) were reduced in the hippocampus and cerebral cortex in pharmacological and genetic models [237, 238, 241, 248, 249, 254–256]. It should be noted that some studies demonstrated no change in CAT and SOD activity in whole brain lysates in Wistar rats in the streptozotocin model [240], while enhanced SOD, GPx, and GR were observed in the mouse cerebral cortex and hippocampus following i.c.v. Aβ_1-42_ injection [247]. It is also important to mention that in transgenic models, the changes depend on animal age. For example, APPswe/PS1dE9 mice at 2.5 months of age did not exhibit any significant changes in measures of OS and cognitive function, whereas 3.5-month-old mice showed diminished SOD and GPx activity and increased MDA and PC levels that were accompanied by spatial memory impairments [255].

### Pharmacotherapeutic Strategy to Reduce OS

Several preclinical studies on AD have shown that many antioxidants can both diminish OS and improve cognitive impairments (Table 11). Among different compounds of special interest are vitamin E, vitamin C, and α-lipoic acid. Vitamin E given 7 days before Aβ_1-42_ decreased MDA and protein carbonyls in the mouse hippocampus and cortex [247]. Similarly, α-lipoic acid enrichment decreased HNE levels in AbPP Tg2576 mouse brains but did not decrease 3-NT levels [251]. In AbPP Tg2576 mice that overexpress a mutant form of APP (beta amyloid (βA), an (A4) precursor protein) and show impaired learning, an R-α-lipoic acid-enriched diet, administered for 10 months, decreased HNE levels in total brain homogenates and also attenuated HNE protein adducts that accumulated around amyloid deposits in the hippocampal and cortical region, but it had little effect on cognitive performance and brain Aβ load. This latter study seems to suggest that a long-term antioxidant therapy that reduced oxidative modifications provided a limited benefit [251].

**Table 11:** Studies with substances/drugs with antioxidant properties in different AD animal models and their influence on oxidative damage and anti-oxidative defense and biomarkers

Model	Oxidative damage and defense biomarkers	Drug and route of administration	Changes in oxidative defense biomarkers	Reference
Aβ_1–42_ i.c.v. to mice (Chinese Kun Ming)	↑MDA, ↓SOD, ↓GPx, ↓GSH in HIP and cerebral CTX	Schisantherin A 0.1 mg/kg for 5 days i.c.v., injection started after 3 days from Aβ_1–42_ injection	↓MDA in cerebral CTX, ↑SOD, ↑GPx, ↑GSH in HIP and cerebral CTX	[241]
Aβ_1–42_ i.c.v. to mice (C57BL/6)	↑MDA, ↑PC, ↑Mn-SOD, ↑Zn, Cu-SOD, ↑GPx, ↑GR in cerebral CTX and HIP	Vitamin E 150 mg/kg, p.o. for 27 days, administration began 7 days before Aβ_1–42_ i.c.v.	↓MDA, ↓PC, ↓Mn-SOD, ↓Zn, Cu-SOD, ↑GPx, Ø GR in cerebral CTX and HIP	[247]
AF64A (a cholinotoxin) 2 nmol/2 μl, bilaterally i.c.v. to rats (Wistar)	↑MDA in HIP	Piperine 5 or 10 mg/kg p.o. 2 weeks before and 1 week after AF64A	↓MDA in HIP	[239]
Streptozotocin 2.57 mg/kg i.c.v. to mice (Swiss albino)	↑TBARS, ↓GSH, ↓GPx, ↓GR in HIP	S-allyl cysteine 30 mg/kg i.p. for 15 days pre-treatment before streptozocin	↓TBARS, ↑GSH, ↑GPx, ↑GR in HIP	[250]
Scopolamine 1 mg/kg i.p single injection to mice (Swiss) 20 min before pretest (memory acquisition) or 15 min after pretest (memory consolidation)	↑MDA, ↓SOD, ↓GPx, ↓GR in CTX and HIP	Imperatorin 1, 5, or 10 mg/kg i.p. 2×/day for 7 days (on 7th day (pretest):10 min before scopolamine injection or 15 min after scopolamine injection	↓MDA, ↑SOD in CTX and HIP, ↑GPx in CTX and HIP, ↑GR in CTX	[248]
Mice (AbPP Tg2576)	↑HNE, ↑3-NT in brain homogenates	α-lipoic acid 30 mg/kg/day enriched diet for 10 months	↓HNE, Ø 3-NT in brain homogenates	[251]
Mice (APP/PSEN1 transgenic)	↑MDA in HIP	Vitamin C 125 mg/kg i.p. for 12 days	Ø MDA in HIP	[252]
Mice (Gulo−/− APP/PSEN1)	MDA level in CTX on vitamin C content standard diet (0.33 g/L of drinking water) not changed	Vitamin C- low diet content 0.099 g/L of drinking water	↑MDA in CTX	[257]
Mice (APPswe/PS1)	nd	Melatonin 5 mg/kg p.o. for 5.5 months	↓MDA, ↓PC in HIP	[260]
Mice (heterozygote APP)	↑TBARS, ↓GSH, ↓SOD in the brain homogenate	Melatonin 10 mg/kg/day for 4 months intargastrically	↓TBARS, ↑GSH, ↑SOD in the brain homogenate	[254]
Mice (APPswe/PS1)	nd	Long-lasting incretin hormone analogue d-Ala^2^GIP 35 days at 25 nmol/kg i.p. once daily	↓8-OHG in CA1 HIP region (in 12 and 19 months old mice)	[258]
Mice (APPswe/PS1)	↑H_2_O_2_, ↑MDA, ↓GSH, ↓TAC in whole brain lysate	Hesperidin 100 mg/kg per day for 16 weeks in chow	↓H_2_O_2_, ↓MDA, ↑GSH, ↑TAC in whole brain lysate	[259]

↑ increase, ↓ decrease, *Ø* no changes, *3*-*NT* 3-nitrotyrosine, *CAT* catalase, *CTX* cortex, *d*-*Ala* ^*2*^ *GIP* glucose-dependent insulinotropic polypeptide, *GPx* glutathione peroxidase, *GR* glutathione reductase, *GSH* glutathione, *GSSG* oxidized glutathione, *HIP* hippocampus, *HNE* 4-hydroxynonenal, *i.c.v*. intracerebroventricular, *i.p*. intraperitoneal, *MDA* malonyldialdehyde, *Mn*-*SOD* manganese superoxide dismutase (located in mitochondria), *PC* protein carbonyl, *SOD* superoxide dismutase, *TAC* total antioxidant capacity, *TBARS* thiobarbituric acid reactive substances, *Zn, Cu-SOD* copper/zinc superoxide dismutase (located in cytoplasm)

In contrast to the study that used α-lipoic acid, vitamin C, when administered to other transgenic lines, such as APP/PSEN1 mice, did not decrease enhanced MDA levels in the cortex or Aβ plaque deposits in the cortex and hippocampal regions in either middle-aged or aged animals [252], although the drug administration improved memory, according to tests that suggested that cognitive rescue was achieved, to some degree, even in animals that suffered from severe neuropathology. The lack of effect of vitamin C on Aβ plaque deposits seems to result from the late introduction of medication in this test because Aβ plaques, considered an end point in the disease process, are detectable in these mice at 4–5 months, which was before the beginning of the test [252]. It is also possible that ascorbate had an effect on soluble Aβ [252]. Reduced vitamin C supplementation has been shown to cause enhanced OS in the form of MDA in APP/PSEN1 mice, which are not able to endogenously synthesize vitamin C [257]. This latter observation led to the conclusion that vitamin C may not be an anti-OS medication per se, but its deficiency in AD patients may lead to oxidative damage. Interestingly, another study showed that the long-lasting incretin hormone analogue d-Ala^2^GIP (glucose-dependent insulinotropic polypeptide) was able to decrease OS biomarkers (i.e., 8-OHG) and amyloid plaque load in 12- and 19-month-old APPswe/PS1 mice [258]. Many natural compounds that possess antioxidant properties have been tested in animal models as AD treatments. Imperatorin and hesperidin diminished brain damage due to OS, and most of them enhanced the power of oxidative defenses [238–241, 259]. Moreover, meloxicam (an anti-inflammatory drug) and selegiline, given alone or in combination, inhibited lipid peroxidation, prevented a decrease in CAT activity, and showed memory-enhancing capacity in a scopolamine AD model [249]. Another compound, *S*-allyl cysteine, which is a sulfur-containing amino acid that was reported to have antioxidant and neurotrophic activity, prevented cognitive and neurobehavioral impairments, prevented ROS damage in the hippocampus, and augmented endogenous antioxidant enzymes in a streptozocin AD model [250]. Similar results were obtained when melatonin was given chronically to a genetic AD mouse model, as the drug alleviated OS and enhanced GSH levels [254, 260]. Moreover, results from Feng et al. [254] showed that OS is an early event in AD pathogenesis and that antioxidant therapies may be beneficial if given at this stage of the disease [254].

As shown above, results from animal AD models that have used various pharmacological compounds to reduce OS and to alleviate memory deficits in AD are promising but do not yet parallel the results obtained in clinical trials.

### Anti-Alzheimer’s Disease Drugs that Modulate Oxidative Balance

Medications used to treat Alzheimer’s disease have been shown to have antioxidant properties that depend on the dose and AD model (Table 12). For example, tacrine, the first anticholinesterase inhibitor approved by the Food and Drug Administration (FDA), was shown to suppress OS in an animal AD model [261]. In fact, tacrine (50–800 μg/kg i.m.) increased the FRAP value, which serves as a measure of “antioxidant power” [262], without elevating any marker of OS-associated damage in brain tissue. The effect of tacrine may therefore be considered to be positive when this drug is used in doses that stimulate the antioxidant system without inducing oxidative damage in brain tissue [263].

**Table 12:** Trials with anti-Alzheimer drugs in different AD animal models and their influence on oxidative damage and anti-oxidative defense biomarkers

Model	Oxidative defense biomarkers	Drug and route of administration	Changes in oxidative defense biomarkers	Reference
Scopolamine 2 mg/kg i.p. once per day for 2 weeks to mice (Kun Ming)	↑MDA, ↓SOD, ↓GSH in HIP	Donepezil (3 mg/kg/day p.o. once per day for 2 weeks)	↓MDA, ↓SOD, ↑GSH in HIP	[238]
Aβ_1–42_ 3 μl of 1 mg/ml solution. i.c.v. to mice (Chinese Kun Ming)	↑MDA, ↓SOD, ↓GPx, ↓GSH in HIP and cerebral CTX	Donepezil (0.01 mg/kg/day i.c.v. for 14 days)	↓MDA, ↑GSH, ↑GPx, Ø SOD in HIP and cerebral CTX	[241]
Mice APPswe/PS1 (transgenic model)	↑H_2_O_2_, ↑MDA, ↓GSH, ↓TAC	Donepezil (2.5 mg/kg/day for 16 weeks)	↓H_2_O_2_, ↓MDA, ↓GSH, Ø TAC	[259]
Scopolamine 1.4 mg/kg i.p. once a day for 9 days to mice (Swiss)	↑MDA, ↓CAT in whole brain lysate	Donepezil (5 mg/kg p.o. once a day for 9 days before scopolamine administration)	↓MDA, ↑CAT in whole brain lysate	[249]
Streptozotocin 0.5 mg/kg i.c. on 1st and 3rd day to mice (Swiss albino)	↑MDA, ↓GSH in whole brain lysate	Tacrine (5 mg/kg/day p.o.) or donepezil (5 mg/kg/day p.o.) for 7 days	↓MDA, ↑GSH (not significant) in whole brain lysate	[261]
Colchicine 5 μg/5 μl i.c.v. injection to rats (Wistar)	↑MDA, ↓GSH in brain	Rivastigmine (2.5 mg/kg p.o. for 28 days started 7 days before colchicine injection)	Ø MDA, Ø GSH in brain	[264]
Kainic acid (KA) 0.4 μg/2 μl single unilateral intrahippocampal injection to rats (Wistar)	↑MDA, ↑nitrate, ↓GSH, ↑GSSG in HIP	Galantamine (2.5 mg or 5 mg/kg for 14 days starting from the day of KA injection)	↑MDA, ↑nitrate, ↓GSH, ↑GSSG in HIP	[266]
Αβ_25–35_ i.c.v. injection once a day for 3 consecutive days to rats (Wistar)	↑iNOS in HIP	Memantine (5 mg/kg/day i.p. for 8 days, 3 times after Αβ_25–35_ injection and for 5 following post-Αβ_25–35_ days)	↓i NOS in HIP	[268]
Age-induced memory impairment in rats (Wistar) (24 months old)	↑PC in HIP and CTX	Memantine (20 mg/kg i.p. for 21 days)	↓PC in HIP and CTX	[267]
Streptozocin 10 μl injection of 3 mg/kg bilaterally on 1st and 3rd day to rats (Sprague-Dawley)	↑ROS, ↑nitrate in CTX and HIP	Memantine (10 mg/kg p.o. for 13 days starting from STZ injection)	↓ROS, ↓nitrate in CTX and HIP	[269]
Kainic acid 200 ng/10 μl bilaterally i.c.v. to rats (Sprague-Dawley)	↑ROS, ↑MDA, in STR, CTX, cerebellum, HIP, ↑nitrite in CTX, HIP	Memantine (10 mg/kg p.o. for 13 days starting from the day of OKA injection)	↓ROS, ↓MDA in STR, CTX, cerebellum, HIP, ↓nitrite in CTX, HIP	[270]

↑ increase, ↓ decrease, *Ø* no changes, *nd* not determined, *3*-*NT* 3-nitrotyrosine, *AlCl*_*3*_ aluminum chloride, *CAT* catalase, *CTX* cortex, *GPx* glutathione peroxidase, *GR* glutathione reductase, *GSH* glutathione, *GSSG* oxidized glutathione, *HIP* hippocampus, *HNE* 4-hydroxynonenal, *i.c*. intracerebral, *i.c.v*. intracerebroventricular, *i.p*. intraperitoneal, *KA* kainic acid, *MDA* malonyldialdehyde, *PC* protein carbonyl, *SOD* superoxide dismutase, *STR* striatum, *TAC* total antioxidant capacity, *TBARS* thiobarbituric acid reactive substances

Donepezil is another cholinesterase inhibitor used in AD patients that, when given in doses as low as 3 mg/kg [259], 5 mg/kg [238, 249], or even 0.01 mg/kg [241] in a mouse AD model, both increased antioxidant power (CAT, SOD, GSH, or GPx, depending on the dose; see Table 12) and diminished lipid peroxidation [238, 241, 249]. However, donepezil, when given in a similar dose of 2.5 mg/kg, failed to combat OS biomarkers and to stimulate antioxidant defenses in the APPswe/PS1 transgenic mouse AD model [259]. Those contradictory results come from studies using non-transgenic and transgenic animal AD models, which means that the multiple adaptations developed for use in these transgenic animals could be the reason for the observed difference in outcomes.

Another medication used in AD treatment is rivastigmine. This drug neither attenuated lipid peroxidation nor restored GSH depletion in the brains of rats in an AD model [264], although an older study indicated antioxidant properties for rivastigmine when AD was induced in rats by aluminum chloride administration [265]. Such differences in the effects of rivastigmine might be caused either by differences in the AD model used in the study (aluminum chlorate p.o. vs. colchicine i.c.v. models) or by differences in the rivastigmine dose regimen (0.3 mg/kg for 3 months vs. 2.5 mg/kg p.o. for 28 days). Based on the above scant reports, it is too soon to either confirm or exclude rivastigmine as an effective OS scavenger in AD.

A single report showed the ability of another AChE inhibitor, galantamine, to reduce OS. In a cognitive impairment animal model, galantamine decreased lipid peroxidation, nitrate, and GSSG levels, enhanced SOD activity, and impaired GSH levels following kainic acid intrahippocampal injection, and it restored cognitive deficits as well [266].

Memantine has also been widely studied in preclinical AD models. For example, it was shown that memantine reduced oxidative damage to proteins in the cortex and hippocampus but not in the striatum, resulting in the reversal of concomitant age-induced recognition memory deficits in aged rats [267]. Other studies found that memantine diminished the level of inducible forms of NOS in an Αβ_25–35_ AD model [268] and ROS and nitrate levels in the hippocampus and cortex in a streptozotocin AD model [269] and in a kainic acid-induced model of dementia [270]. However, memantine was shown to have neuroprotective properties not only in AD models but also in 3-nitropropionic acid [271], rotenone [272], and diisopropylphosphorofluoridate (DFP) toxicity models [273].

There is a wide range of evidence showing that several drugs used to treat AD have antioxidant properties, suggesting that at least part of their efficacy in animal models may come from that action.

## Summary and Conclusions

In general, the presence of OS in the pathophysiology of many neurodegenerative disorders, including ALS, PD, and AD, is a well-recognized phenomenon. The results of many in vitro and in vivo preclinical and clinical studies have consistently demonstrated that OS is one of the crucial players in the degeneration that occurs in the nervous system. The imbalance between OS and antioxidant defense systems seems to be a universal condition in neurodegeneration. However, what can be surprising is that the results of many studies often provide different results when trying to determine the exact mechanisms that underlie OS and to determine which of the markers of OS could be clinically useful. What has been shown to be elevated in one study does not necessarily have to rise in another. In preclinical studies, these divergent results could be explained by the use of different models, different species, or different methodologies. As for the clinical setting, it must be stressed that the number of patients available for study is usually small because they are in different stages of their diseases, there are often coexisting comorbidities, and, last but not least, they often take many other medications with different pro- or antioxidant properties. The analysis of potential biomarkers under these conditions is extremely difficult. Therefore, assessing the real efficacy of potential antioxidant drugs is a challenge. However, there are some data, if even modest, that some of the existing drugs possess anti-oxidant properties and that they could slow down neurodegenerative processes and improve our understanding of the significance of OS in the pathobiology of these untreatable conditions.

The results of clinical and preclinical studies have demonstrated the presence of elevated levels of OS biomarkers as well as impairments to antioxidant defenses in the brain and peripheral tissues in PD, AD, and ALS. As the currently available therapies for these neurodegenerative diseases are not sufficiently effective for treating disease symptoms, novel substances are searched for. Among these, drugs with antioxidant activity, which are widely studied as a possible anti-neurodegenerative PD, AD, or ALS agents, can efficiently normalize biomarkers of the oxidant/antioxidant balance in animal models. Most such drugs have so far failed to slow down the progression of the disease or to prolong the lives of patients. Some exceptions within these anti-neurodegenerative drugs exist, and they give hope and inspire further research.

## Abbreviations

3-NT: 3-Nitrotyrosine
4-HDA: 4-Hydroxyalkenal
5-OHC: 5-Hydroxycytosine
5-OHU: 5-Hydroxyuracil
6-OHDA: 6-Hydroxydopamine
^62^Cu-ATSM: Copper-diacetyl-bis(N4-methylthiosemicarbazone
8-OHA: 2,8-Hydroxyadenine
8-OHdG: 8-Hydroxy-2′-deoxyguanosine
8-OHG: 8-Hydroxyguanine
AD: Alzheimer’s disease
AGE: Advanced glycation end product
ALE: Advanced lipid peroxidation end product
ALS: Amyotrophic lateral sclerosis
AOPP: Advanced oxidation protein products
APOE: Apolipoprotein E
APP: Amyloid precursor protein
ATP: Adenosine triphosphate
Aβ: Beta amyloid
CAT: Catalase
COMT: Catechol-*O*-methyltransferase
COX: Cyclooxygenase
CSF: Cerebrospinal fluid
CTL: Creatol (5-hydroxycreatinine)
CTX: Cortex
CYP 450: Cytochrome P450
DHE: Docosahexaenoic acid
eNOS: Endothelial nitric oxide synthetase
EPA: Eicosapentaenoic acid
ESR: Electron spin resonance
ETC: Electron transport chain
F2-isoPs: F2-isoprostanes
F4-NP: F4-neuroprostane
FALS: Familial amyotrophic lateral sclerosis
FDA: Food and Drug Administration
GPx: Glutathione peroxidase
GPx1: Glutathione peroxidase-1
GR: Glutathione reductase
GSH: Glutathione
GSH/GSSG: Reduced/oxidized glutathione ratio
GSSG: Oxidized glutathione
GSSG/GSH: Oxidized/reduced glutathione ratio
GST: Glutathione *S*-transferase
HD: Huntington disease
HETE: Hydroxyeicosatetraenoic acid
HIF-1a: Hypoxia-inducible factor-1a
HIP: Hippocampus
HNE: 4-Hydroxynonenal
HODE: Hydroxyoctadecadienoic acid
iNOS: Inducible nitric oxide synthetase
i.c.v.: Intracerebroventricular
i.p.: Intraperitoneal
i.v.: Intravenous
Intra-SNc inj.: Intra-substantia nigra injection
Intra-STR inj.: Intrastriatal injection
IsoF: Isofuran
IST: Immuno-spin trapping technique
LF: Lipofuscin
LOX: Lipoxygenase
LPO: Lipid hydroperoxide
Lyso PC: Lysophosphatidylcholine
MAO: Monoaminooxidase
MAO-B: Monoamine oxidase B
MDA: Malondialdehyde
MPO: Myeloperoxidase
MPTP: 1-Methyl-4-phenyl-1,2,3,6-tetrahydropyridine
NADH: Reduced form of nicotinamide adenine dinucleotide
MRI: Magnetic resonance imaging
NADPH: Reduced form of nicotinamide adenine dinucleotide phosphate
NF: Neurofuran
nNOS: Inducible nitric oxide synthetase
NOX: NADPH oxidase
ONOO^−^: Peroxynitrite
NS: Nitrosative stress
Opht A: Ophthalmic acid
ox-LDL: Oxidized LDL
OS: Oxidative stress
p.o.: Per os
PC: Protein carbonyl
PD: Parkinson’s disease
PET: Positron emission tomography
Prx: Peroxiredoxin
Prx2: Peroxiredoxin-2
PSEN 1: Gene encoding presenilin 1
RIA: Radioimmunoassay
RNS: Reactive nitrogen species
ROS: Reactive oxygen species
s.c.: Subcutaneous
SAG: Superoxide anion generation
SALS: Sporadic amyotrophic lateral sclerosis
SN: Substantia nigra
SNpc: Substantia nigra pars compacta
SOD: Superoxide dismutase
SOD1: Copper/zinc superoxide dismutase
STR: Striatum
TAC: Total antioxidant capacity
TBARS: Thiobarbituric acid reactive substances
TQ/TQH2: Tocopherylquinone/tocopheryl hydroquinone
Trx: Thioredoxin
TSE: Aqueous extract of tomato seeds
TT: Total thiol
Tyr: Tyrosine
UQ/UQH2: Ubiquinone/ubiquinol
XO: Xanthine oxidase
ω-3 FA: Omega-3 fatty acid
